# Establishing Suitable Bra Characteristics for Tactical Athletes: A Mixed-Method Multi-Study Approach

**DOI:** 10.1007/s40279-025-02375-3

**Published:** 2025-12-15

**Authors:** Emily Paines, Gemma Milligan, Mike Tipton, Andrew Roberts, Alex J. Rawcliffe, Jenny Burbage

**Affiliations:** 1 School of Psychology, Sport and Health Sciences, Research Group in Breast Health, University of Portsmouth, Spinnaker Building, Cambridge Road, Portsmouth, PO1 2ER UK; 2 Army Recruit Health and Performance Research, Medical Branch, HQ Army Initial Training Command, Ministry of Defence, Andover, UK

## Abstract

**Background:**

A professional sports bra fitting and issue service was introduced for women entering British Army basic training (BT) in 2020 to address breast health and bra-related issues. However, the suitability of commercial off-the-shelf sports bras for female tactical athletes, designed primarily for short-duration use, remains unclear.

**Objective:**

We aimed to develop evidence-based recommendations to inform British Army sports bra policy and establish a framework applicable to other female tactical athlete populations.

**Methods:**

A mixed-method multi-study approach was employed (May 2021– September 2023). First, a cross-sectional study was conducted with BT recruits to assess the bra fitting and issue service using questionnaires (*n* = 244) and semi-structured interviews (*n* = 7). A concurrent task analysis with subject matter experts (*n* = 8) identified BT activities that were both physically demanding and challenging for the breast. Second, a controlled laboratory study with recruit-matched civilians (*n* = 25) examined the performance of various sports bra characteristics during short-duration simulations of military-specific tasks. Finally, a 14-week longitudinal field study of BT recruits (*n* = 93) monitored sports bra performance during sustained wear, enabling comparisons between laboratory-based simulations and real-world use.

**Results:**

Despite implementing a bra fitting and issue service, 61% of recruits still reported at least one breast or bra-related issue. None of the four sports bra designs tested fully met the varied demands of BT tasks. Ten key bra design characteristics (e.g. strap configuration, ease of use, support level) were identified across five different BT tasks (physical training, field exercise, military tasks, foot drill and classroom sessions), combining insights from short-duration laboratory simulations and long-duration field use.

**Conclusions:**

These evidence-based recommendations can enhance breast health, comfort and performance in female military recruits. Findings have broader implications for female tactical athletes in physically demanding occupations, supporting the development of optimised female-specific equipment.

**Supplementary Information:**

The online version contains supplementary material available at 10.1007/s40279-025-02375-3.

## Key Points

**Table:** 

Current sports bras do not fully meet the physical demands of British Army basic training for women.
Clear design features have been identified that can reduce discomfort and support women during physically demanding tasks.
Findings highlight the urgent need for a tactical bra designed for arduous occupational roles, with a framework that can be applied to improve provisions for female tactical athletes worldwide.

## Introduction

Tactical athletes perform in service occupations such as the military, law enforcement and firefighting, and require high levels of physical fitness to perform their duties, often in stressful and potentially life-threatening situations [1]. The physical demands of tactical athletes vary according to their specific occupation and role, necessitating evidence-based approaches to optimise clothing and equipment for operational effectiveness [1]. In the UK alone, there are an estimated 71,735 female tactical athletes [2–4]. Breast support has been identified as a critical component of individual equipment for female military personnel, with many nations prioritising the development of female-inclusive clothing to enhance diversity and performance [5]. In 2016, the UK lifted its ban on women serving in British Army ground close combat roles, setting a target to increase female representation to 30% by 2030 [6]. As efforts continue to increase female recruitment, addressing female-specific health and well-being concerns (such as breast health) will be essential for improving performance and retention in military service and across wider tactical athlete populations.

Breast and bra-related issues are widely reported as barriers to physical activity, with concerns including difficulty finding a well-fitted and appropriate sports bra, embarrassment because of excessive breast movement, breast pain and breast size [7–10]. British Army basic training (BT) is a physically demanding process designed to transform civilians into trained soldiers. An investigation into breast and bra issues among female BT recruits found that 75% experienced at least one breast-related concern [11]. This study identified sports bras as a necessary item of equipment to increase comfort and reduce breast movement, yet women found it difficult to source well-fitted sports bras and demonstrated poor breast health knowledge. Issuing sports bras to BT recruits significantly reduced reports of excessive breast movement, although there was an increase in some bra fit issues (such as straps and the underwire digging in) [11]. In response to these findings, an enduring professional sports bra fitting and issue service (BraFIS) was introduced in January 2020 for all female British Army recruits (c. 1200 recruits use the BraFIS per year).

Sports bras have traditionally been designed for short duration high-intensity activities such as running [12–14]. Sports bra performance is typically assessed using metrics such as the reduction in breast movement, breast displacement and strap pressure [15]. Existing research has examined various design characteristics that impact sports bra performance, including underwire presence, strap adjustability and configuration, principle fibre content, underband adjustability, closure type and location, cup padding and neck drop [15–18]. Previous research has focused predominantly on running activities, with limited evaluation of whether issued sports bras meet the diverse demands of BT (e.g. load carriage, crawling, jumping and weapon handling).

Military tasks impose unique physiological and biomechanical challenges, including prolonged wear, load carriage, and interaction with equipment such as Bergens (large military rucksacks) and body armour. While Choi-Rokas et al. [19] examined subjective perceptions of fit, mobility, and comfort in US Army personnel, their study lacked objective measures of breast support. Key considerations such as thermal burden and hygiene, relevant in extended and high-heat use, remain unexplored. Notably, 37% of BT recruits are classified as larger breasted [11], a factor associated with increased breast motion, pain and different bra preferences [7, 12, 20–24]. While Burbage et al. [11] provide insights into sports bra usage and preferences in BT, they do not differentiate experiences by breast size, an important consideration for optimising the BraFIS. A more holistic investigation is required to determine suitable bra characteristics for the tactical athlete and addressing these gaps will be critical to ensure that sports bras address both performance and comfort in demanding occupational settings.

Using a novel and rigorous approach, the current research aimed to examine the bra requirements of female British Army recruits in BT, and to develop evidence-based recommendations for improving the existing BraFIS, establishing a framework that can be utilised across other female tactical athlete populations. Specifically, *Study 1* evaluated the existing BraFIS and determined the most physically and breast-demanding tasks in BT. *Study 2* investigated four commonly issued sports bras (both objectively and perceptually) during short duration laboratory simulations of BT tasks. *Study 3* investigated the same sports bras (as Study 2) over the full duration of BT (14 weeks) to determine their suitability for long-duration use.

## General Methods

**Fig. 1:**
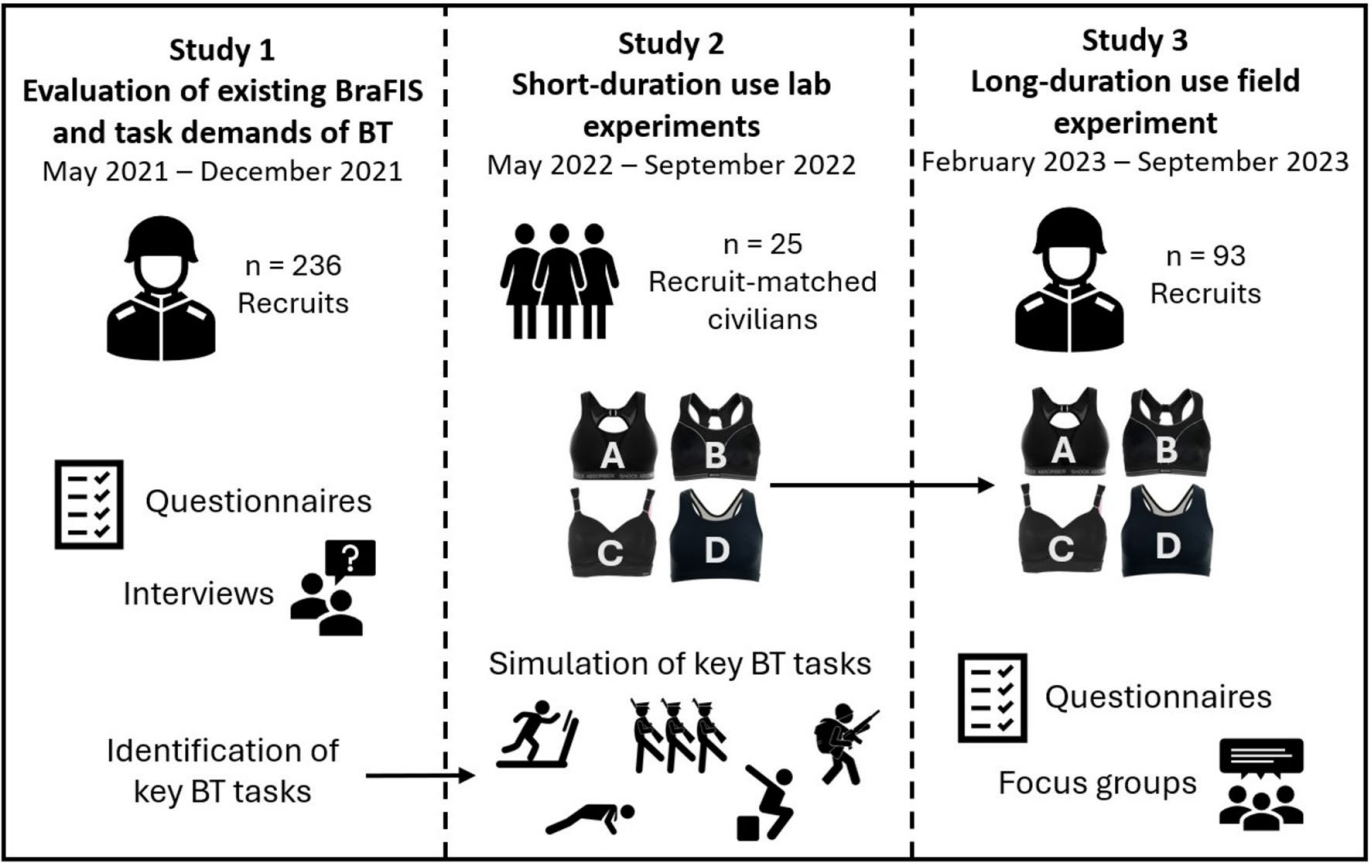
Overview of study progression: (Study 1) evaluation of existing sports bra fitting and issue service (BraFIS) and task demands of basic training (BT); (Study 2) short-duration laboratory testing of four sports bras during simulated BT tasks; and (Study 3) long-duration field evaluation of bras during BT

This research programme consists of three interconnected studies (Fig. 1) that adopted a mixed-methods approach to address both the physical demands of BT and the specific demands placed on the breasts, culminating in a comprehensive evaluation of issued sports bras and the BraFIS. The multi-modal approach used has been reported to enhance the validity of the data collected owing to the variety of collection methods used [25, 26]. All statistical analyses were undertaken in the IBM SPSS 26 statistics package with an alpha level of 0.05.

### Demographic Data

Different participants were used in each study (Table 1) although participants had similar characteristics, including body mass index (kg m^−2^) and bra size. Breast mass was estimated from the cup and underband size using the method proposed by Turner and Dujon [27], where 115 g per cup size for underband sizes of 32–34 inches, and 215 g for underband sizes of 36–38 inches were used. Participants were then categorised into smaller breasted (< 500 g) and larger breasted (> 500 g) groups [28]. For participants with underband sizes of 28–30 inches, a cross-grading system was applied [20, 29]. Participants who were pregnant or had been pregnant or breast fed in the previous year were excluded.

**Table 1:** Mean ± SD participant demographics across Study 1 (evaluation of existing sports bra fitting and issue service and task demands of Basic Training; *n* = 236), Study 2 (short-duration use laboratory experiments; *n* = 25) and Study 3 (long-duration use field experiment; *n* = 93)

Mean ± SD	Study 1 (*n* = 236)	Study 2 (*n* = 25)	Study 3 (*n* = 93)
Age (years)	21 ± 4	25 ± 5	22 ± 4
Height (m)	1.66 ± 0.1	1.65 ± 0.1	1.65 ± 0.1
Body mass (kg)	63.6 ± 8.3	67.3 ± 9.8	66.2 ± 10.7
BMI (kg·m^−2^)	23.1 ± 2.7	24.9 ± 3.6	24.3 ± 3.3
Bra size^a^ (UK underband and cup size)^b^	34B	34B/34C	34C/34D

*BMI* body mass index, *SD* standard deviation

^a^Bra size is self-reported in studies 1 and 3, bra size was determined with professional bra fitting in study 2

^b^Modal/bimodal data presented

### Questionnaires for Study 1 and 3

Limesurvey™ was used to disseminate questionnaires that participants completed on their own personal devices. Questionnaires were piloted by BT recruits (Study 1, *n* = 9; Study 3, *n* = 13), who represented the target population. Piloting assessed readability, length and appropriateness of the questionnaire prior to full distribution. Questionnaires combined multiple-choice, scale (1–10), Likert (1–5) and free-text questions, designed for a military population based on previously validated questionnaires [11, 22, 30].

### Bras Chosen for Study 2 and 3

**Fig. 2:**
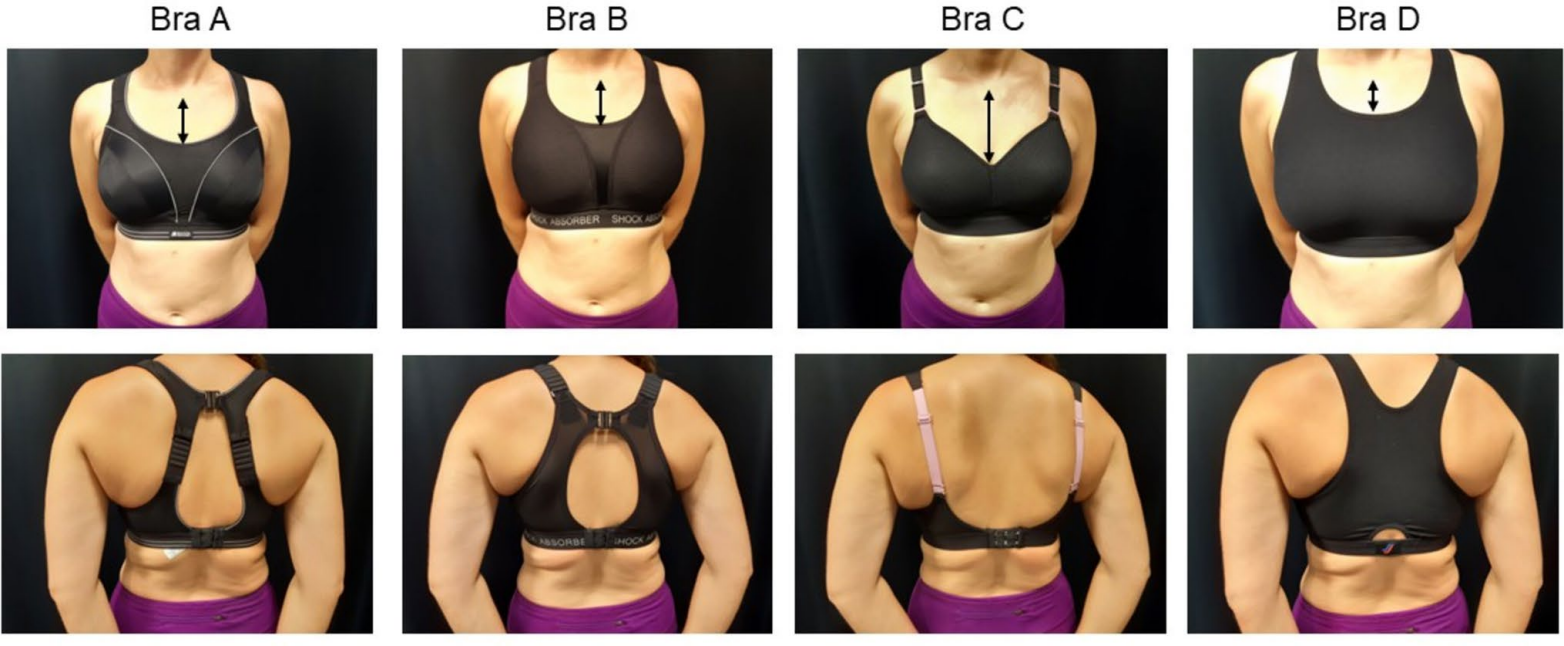
Anterior and posterior view of sports bras A (Shock Absorber Padded Run), B (Shock Absorber S5044), C (Triumph Hybrid Lite) and D (Sportjock Super/Action). Model size 34C. *Black arrows* on the model represent the location the neck drop distance was measured (ESM)

Four commonly issued sports bras by the BraFIS were selected for Studies 2 and 3; these encompassed a wide range of characteristics (Electronic Supplementary Material [ESM]), which were previously identified to contribute to performance [15]. Neck drop was calculated by measuring the distance (cm) from the sternal notch to the top of the bra (Fig. 2) on a standardised mannequin. A distance of < 9.9 cm was classed as a *high* neck drop, ≥ 9.9 cm and ≤ 11.6 cm as a *medium* neck drop and > 11.6 cm was a *low* neck drop [15].

## Study 1 (Evaluation of Existing BraFIS and Task Demands of BT)

### Methods

#### Study Design and Participants

This study used a cross-sectional qualitative observational design with a recruit questionnaire at the end of BT (ESM). Semi-structured interviews were conducted with subject matter experts (SMEs; members of training staff) and recruits (ESM). Training manuals were then reviewed to determine the frequency and duration of tasks identified by SMEs [31].

Female British Army recruits undertaking BT were briefed by the study team and subsequently invited to take part in the study during the last 2 weeks of their training. The sample size for the questionnaire (a minimum of 226) was based on an a priori power calculation (G*Power) for *Χ*^2^ analysis with a medium effect size (0.3), an alpha level of 0.05 and a power of 0.8. A minimum of 87 participants in each of the breast size groups allowed for an analysis between groups. A total of 244 participants completed the questionnaire, over an 8-month period (May–December 2021). Eight questionnaires were returned uncompleted and therefore were excluded, leaving 236 completed questionnaires for analysis (Table 1). A total of eight SMEs (six female individuals and two male individuals) and seven recruits were interviewed. All SMEs were required to have met at least one of the six criteria adapted from Blacklock et al. [32] (ESM), which was confirmed by participants at the beginning of the interview.

#### Procedures

During the first week of BT, female participants attended the BraFIS, delivered by the contracted supplier (BoobyDoo Limited^®^), and were issued with three sports bras of their choosing from the range of bras offered. At the end of BT, a 37-item questionnaire, split into four sections (Personal Information, Sports Bra Use and Preferences, Basic Training and Sports Bra Use, Breast History), was distributed.

Training schedules and semi-structured interviews with SMEs (*n* = 8) and recruits (*n* = 7) (conducted via Zoom^®^, lasting up to 90 min) were used to establish the physical demands of BT and obtain individual task descriptions. Details were provided on: typical training days, standard operating procedures, kit and equipment details, task criticality, frequency, duration and physical intensity (e.g. easy, moderate, hard). These data were used to compare the physically demanding tasks of BT with those perceived to be the most demanding on the breast and inform the task simulations used in Study 2.

#### Data Analysis

A descriptive analysis of questionnaires was conducted to summarise recruits’ demographics; the most physically demanding tasks; activities that recruits perceived to require the most breast support; and bra-wearing habits. Chi-squared goodness-of-fit tests were used to assess the association of dependent variables (physically demanding tasks, breast and/or bra issues) between small-breasted and large-breasted groups [7]. No statistics are reported where no significant differences were found between breast size groups. Cramer’s *V* effect sizes were interpreted as small (0.10), medium (0.30) and large (0.50) [33], and 95% confidence intervals were reported. Standardised adjusted residuals were also calculated to determine where these differences occurred, where greater than ± 2 indicates the value is significant at *p* < 0.05 [34].

Audio recordings from recruit and SME interviews were transcribed verbatim, first using Zoom^®^ software followed by a researcher review. Because of limited previous research in this area, an inductive content analysis was conducted to identify, organise and count key themes [35]. Physically demanding tasks highlighted by SMEs were analysed for frequency, duration, distance, load carried, procedures, progressions and fitness components.

### Results

#### Questionnaires

Whilst 62% of recruits wore the issued bras, 38% did not, citing “comfort”, “size” and “preferring their own sports bra” as reasons for not wearing the issued bras. Uptake of issued bras was not associated with breast size (*X*^2^ (1) = 2.793, *p* = 0.095, *V* = 0.111). The BraFIS most frequently issued a mixture of different bra styles to participants (32%), closely followed by compression style only (31%). The style most worn by participants was a compression style (30%). A total of 76% reported wearing a sports bra always (7 days/week) or often (5–6 days/week), with 76% wearing it all day or over 8 h daily; overall, 59% reported not changing their bra during the day (ESM). No significant associations were found between smaller and larger breasted recruits for bra use and preferences (including frequency and duration of use, style of sports bra issued and worn by recruits, and changing habits).

In total, 142 bra issues were reported, with 60% of participants reporting one or more issues with the bras provided (ESM). There was no significant difference in the overall number of issues reported across breast size groups (*u* = 1800, *z* =  − 0.771, *p* = 0.441). The most reported issues were the quantity, size, fit and style of the bras provided. When asked if they could change anything about the sports bras, a total of 91 responses were received (ESM). Participants also reported that breast and bra issues negatively impacted their performance across different BT tasks (ESM). Gym cardio sessions were most frequently affected by *excessive breast movement* ‘all of the time’ (45%). Participants reported that bra *rubbing and chafing* (46%) and *bra straps were digging in* (41%) most affected their loaded march performance. Across all tasks, except classroom sessions, *shoulder straps digging in* negatively affected performance ‘all of the time’ for at least 25% of participants (ESM).

Participants indicated that the sports bras were less comfortable when worn in combination with additional equipment (ESM). The most common themes (frequency of comments [fc]) identified from additional comments about the BraFIS identified by both recruit and SME interviews were *confusion with kit lists and the number of bras to bring to training* (fc = 6), *bra delivery time* (fc = 5), *breast size changes during BT* (fc = 4), *access to bras* (fc = 4) *and education* (fc = 2) [ESM].

#### Task Analysis

Physical training (PT) sessions (inclusive of strength and conditioning lessons and outdoor PT) were most frequently cited as physically demanding by SMEs. Recruits perceived loaded marching and gym cardio to be most physically demanding closely followed by outdoor PT. Overall, recruits reported sports bras to be most needed during: gym cardio (50%); outdoor PT (42%); team sports (36%); fire and movement (35%); gym group sessions (31%); and loaded marching (28%). Sports bras were rated to be least needed during classroom sessions (66%). Foot drill and range activities had a greater spread of data across all scores, with few recruits indicating these tasks to be both least and most needing a sports bra. Summary descriptions of BT tasks are provided in Table 2.

**Table 2:** Summary of BT tasks that were most physically demanding tasks, and most needing a sports bra

Task	Frequency/duration	Equipment/clothing	Task description
**PT sessions** S&C Outdoor PT	9–11 sessions 45–120 min 4–12 sessions 60–120 min	Issued PT kit: trainers, shorts, T-shirt Boots, combats, shirt/U back, helmet (knee pads for obstacle course)	Builds in difficulty throughout the course of BT, encourages learning and development of fundamental movement patterns for strength development, e.g. squats, lunges, burpee, deadlifts and push press Functional movement HIIT training, including crawling, carrying Bergen’s, casualty drags, partner carry and overhead press with Bergen’s Obstacle course: various jumping and landing/rolling activities including: under-over bars, ditch jump, 1.2-m wall, 1.8-m wall, 3.7-m wall (as a team), rope ladder, monkey bars, plank walk with jump and crawling
Loaded march	8–16 sessions 70–120 min	Full kit: boots, combats, shirt/U back, helmet, webbing, body armour, daysack/Bergen, weapon, ammunition Load ranges 10–40 kg	Loaded marching sessions are progressive with distance, speed and load being carried increasing throughout training, up to the requirements of the RFT(BT) RFT(BT): 4-km TAB with 20 kg in 50 min followed by a 2-km TAB with 15 kg in 15 min
Gym cardio (running)	4–5 sessions minimum (exact number is PT staff led dependent on recruit needs) 60–120 min	Usually, PT kit and trainers Occasionally booted runs will take place: boots trousers Shirts/U backs	Gradual increase in distance throughout the course of BT. Around training area, across hills. No more than 5 km. Continuous or intervals based on PTI’s decision
Foot drill	18–34 sessions 60–100 min (extended nearing pass out)	N/A	Military-specific disciplined movements to instruct soldiers in marching, standing, turning and saluting in unison. Featuring exaggerated heel strike and foot stamps

*BT* basic training, *HIIT* high-intensity interval training, *mins* minutes, *PT* physical training, *PTI* physical training instructors, *RFT(BT)* role fitness test (basic training), *S&C* strength and conditioning, *TAB* tactical advance to battle, *N/A* not applicable

## Study 2 (Short-Duration Use Laboratory Experiments)

### Methods

#### Study Design and Participants

Study 2 was an experimental within-participant counterbalanced laboratory study assessing the performance of sports bras using quantitative (e.g. breast biomechanics, skin temperature) and qualitative (e.g. perception of support, comfort) methods. Because of logistical constraints, it was not feasible for BT recruits to attend the laboratory on seven separate occasions over several weeks. Therefore, civilian participants for Study 2 were recruited from the local population via advertisements and word-of-mouth. Civilians were carefully selected to match BT recruits in breast size, age, height and mass. Nonetheless, we recognise that the use of civilians may limit the ecological validity of findings related to military-specific movements. A minimum sample size of 24 was calculated using G*Power software (Dusseldorf, v3.1.7) for a one-way repeated-measures analysis of variance (four factors). This assumed a power of 0.8 and a Cohen’s *d* effect size of 0.7, as observed in similar studies using the same dependent variables for breast biomechanics data [36]. Thirty participants were recruited into the study to allow for drop outs; a total of 25 participants (Table 1) completed the study.

#### Procedures

Upon arrival, participants’ height (Seca Ltd, Leicester, UK) and mass (Model B150S; Sartorius UK Ltd., Epsom, UK) were recorded in gym attire (leggings and t-shirt) to the nearest 0.1 cm and 0.05 kg. Participants were fitted with an everyday bra (M&S, underwire, full cup) using professional criteria [37], which was used to estimate breast mass (Sect. 2.1). Participants undertook a self-selected warm-up on the treadmill (including a familiarisation period if required) whilst wearing their own exercising bra before donning the first of the four randomly assigned bras (Sect. 2.2).

To quantify breast movement during treadmill running, lightweight motion sensors were secured in accordance with standardised methodology for breast biomechanical data collection [15]. Data were collected using a six-degrees of freedom motion sensor system (240 Hz; Liberty Micro Sensor 1.8; Polhemus, Colchester, VT, USA). The treadmill was set to 13 km·h^−1^, approximately 80% of the average maximal aerobic speed of female recruits during BT running sessions (calculated from a BT programme schedule with advice from SME). Participants ran for 30 s on a zero gradient, after which sensor data were recorded for 10 s to capture five gait cycles. The process was repeated in all four bras (Fig. 1) in a counterbalanced order. A fifth bare-breasted trial was conducted to determine the percentage reduction in breast movement.

Participants also completed three simulated military tasks (foot drill, drop landings and burpees) and a loaded march. These activities were selected from tasks identified in study 1 to be demanding on the breast, drop landings and burpees are frequently occurring elements of PT sessions (identified by SMEs). Five repetitions of four British Army foot drills were performed in two sequences: Quick March to Halt and Stand at Ease to Stand at Attention. The foot drills followed the British Army’s drill manual [38] and were taught by an experienced research team member. For drop landings, a height of 0.9 m was chosen as a mid-range between the minimum (0.5-m) and maximum (1.35-m) drop heights in a BT obstacle course, undertaken during outdoor PT sessions. Participants stood on a 0.9-m box with hands on hips during the drop. To reduce injury risk, the landing technique was practiced from 0.3-m and 0.6-m heights during familiarisation. Participants were instructed on performing a standardised burpee, with movement speed set to 60 b·min^−1^ using a metronome, ensuring each of the five burpee stages lasted 1 s. Five burpees were performed consecutively. For each simulation, a Miqus M1 camera (Qualisys, Gothenburg, Sweden) recorded bra movement for a qualitative analysis; quantitative breast movement data were not collected because of the limited capture volume of the Polhemus sensors. Participants rested for 1–2 min between trials and had a 10-min recovery after each bra condition; this was repeated for all four bras.

The loaded march replicated the load carriage lesson from week 6 of the BT programme. Participants completed the protocol in a counterbalanced order, wearing a bra, military t-shirt, trainers and gym shorts/leggings. Before participants donned the loaded march equipment, thermal images of their anterior torso (both bare breasted and in the bra) were taken using a thermal imaging camera (FLIR systems) [39]. They first donned 10 kg of equipment (Bergen, helmet, three 0.1-kg weights and dummy rifle; ESM), then completed a 2-km walk at 4.8 km·h^−1^ for 25 min. After a 5-min rest, the weight was reduced to 7.5 kg (daysack and rifle), and a final 2-km walk for 18 min at 6.6 km·h^−1^ was completed. Thermal images of the anterior torso both bare breasted and in the bra were taken exactly 2 min after the cessation of the loaded march task, allowing time to remove the equipment and t-shirt. Perceptual measures (thermal sensation, comfort, rating of perceived exertion, skin wettedness) and bra mass were recorded to assess non-evaporated sweat loss. Sessions were scheduled at least 7 days apart to allow adequate recovery and minimise potential cross-over effects. After each trial within the simulated tasks detailed above, participants completed a perceptual questionnaire rating breast support, comfort, pain and any issues (e.g. rubbing/chafing) experienced in the bras (ESM). Upon completing all trials, participants completed further questionnaires to rank the suitability of each bra per simulated task and overall (ESM).

#### Data Analysis

All positional data from the electromagnetic sensors were exported into Visual 3D (v2021. 12; HAS-Motion, Kingston, Ontario, Canada) and filtered using a recursive, second-order, low-pass Butterworth filter with a cut-off frequency of 13 Hz [40]. Gait cycles were identified using every other inferior minima of the suprasternal notch sensor [15], the first five complete gait cycles from the treadmill test were identified within the data and used for analysis. Three-dimensional nipple displacement, velocity and acceleration, and percentage breast movement reduction, were calculated using methods from Norris et al. [15].

A qualitative video capture was used for military movements to corroborate comments made by participants within questionnaires. Qualisys Track Manager software was used to collect, process and review the recordings. From thermal images recorded, the average skin temperature of the breasts and bra surface temperature were calculated both pre- and post the loaded march using methods from Ayres et al. [39]. The difference between the bra mass pre and post the loaded march was calculated to indicate the non-evaporated sweat loss and was compared between each bra condition.

Dependent variables were graphically examined for normality prior to the statistical analysis. A series of one-way analyses of variance with post-hoc paired sample *t* tests (with Bonferroni corrections) were used to assess any differences (*p* < 0.05) between bra conditions in measures of breast movement reduction, breast displacement, velocity and acceleration, bra mass, breast skin and bra temperature. Mean differences between significant pairs were presented with corresponding effect sizes (Cohen’s* d* criteria: ≤ 0.2 is considered trivial, 0.21–0.5 is small, 0.51–0.8 is moderate and ≥ 0.8 is large [33]). A non-parametric analysis of perceptual measures of rating of perceived exertion, thermal sensation, thermal comfort and skin wettedness was performed using Friedman’s analysis of variance, post-hoc Wilcoxon signed-rank tests were performed. Pearson’s *r* effect sizes were calculated and interpreted as small (*r* = 0.1), medium (*r* = 0.3) and large (*r* = 0.5) [33].

A descriptive analysis of questionnaires was conducted to summarise participants’ perceptions of bra support, comfort and suitability. Numerical rating scales were used to assess breast support (0 ‘very unsupportive’ to 10 ‘very supportive’) and bra comfort (0 ‘very uncomfortable’ to 10 ‘very comfortable’). Suitability was assessed using a five-point Likert scale (possible answers were ‘very unsuitable’, ‘unsuitable’, ‘neutral’, ‘suitable’ and ‘very suitable’). Performance of bras were ranked after each task and overall from one (best performing) to four (worst performing). In line with similar research [8, 11, 26] inductive content analyses were conducted on free-text answer questions.

### Results

#### Breast Biomechanics

**Fig. 3:**
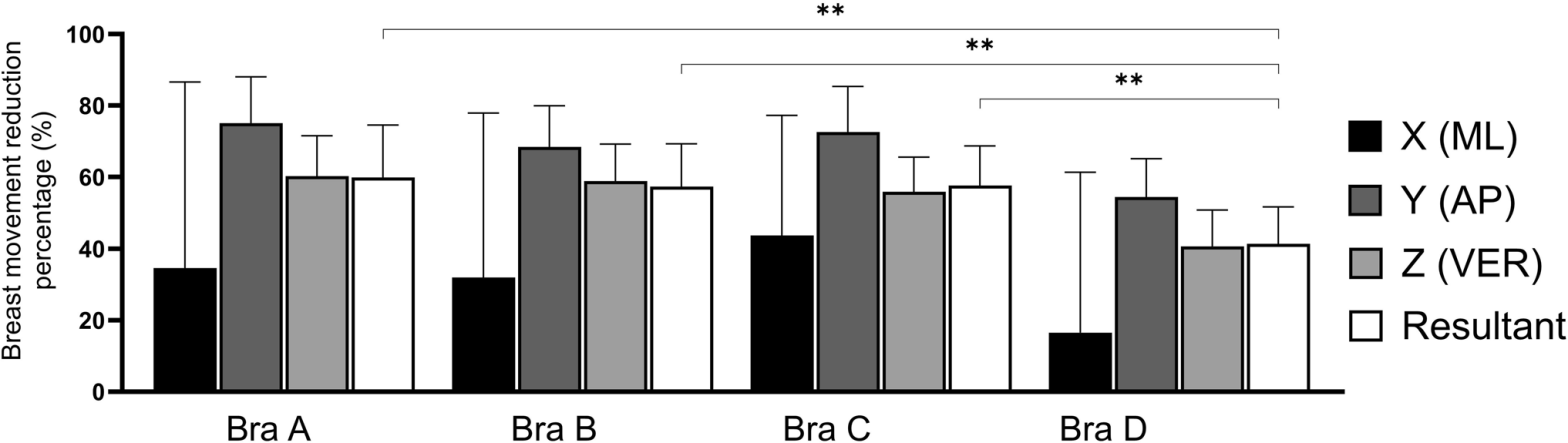
Percentage reduction of breast movement (mean ± standard devitation) of the four sports bras tested during treadmill running (13 km.h.^−1^) [*n* = 25]. *AP* anteroposterior, *ML* mediolateral, *VER* vertical, **represents a significant difference *p* < 0.001

Breast movements were significantly reduced (*F*_(3,72)_ = 77.718,* p* < 0.001) across the four bras (Fig. 3) during treadmill running. A post hoc analysis showed bras A, B and C significantly reduced (*p* < 0.001) breast movement more (with large effect sizes) compared with bra D (*t* = 10.751,* d* = 1.37; *t* = 12.696, *d* = 1.26; *t* = 10.616, *d* = 1.33 respectively). No significant differences and small effect sizes were found between any of the other bras. Overall, a percentage reduction in breast movement results suggest that bra A (62%) was marginally better than bras B (59%) and C (60%). However, bra D (44%) was found to be significantly poorer compared with all other bras in all participants. This finding is confirmed by the breast displacement, peak breast velocity and peak breast acceleration data (see ESM), which demonstrated that bra D performed worst for support.

#### Perceptual Measures

Across all tasks, Bra D was consistently rated as the least supportive bra (ESM), a finding further reinforced by high-speed video footage showing excessive motion. Bra A was rated as the most supportive bra across all tasks; the strap design and compressive and padded cups reported to provide superior support. Despite being the least supportive, Bra D was rated the most comfortable bra for military movements and loading marching. Comfort ratings were however mixed across all bras, with no single bra performing significantly better (ESM). Bras A and B caused the most reports of rubbing and chafing, particularly around the straps and underband. Participants wearing Bra D for the loaded march noted discomfort when carrying the rifle because of the lack of cup padding.

**Fig. 4:**
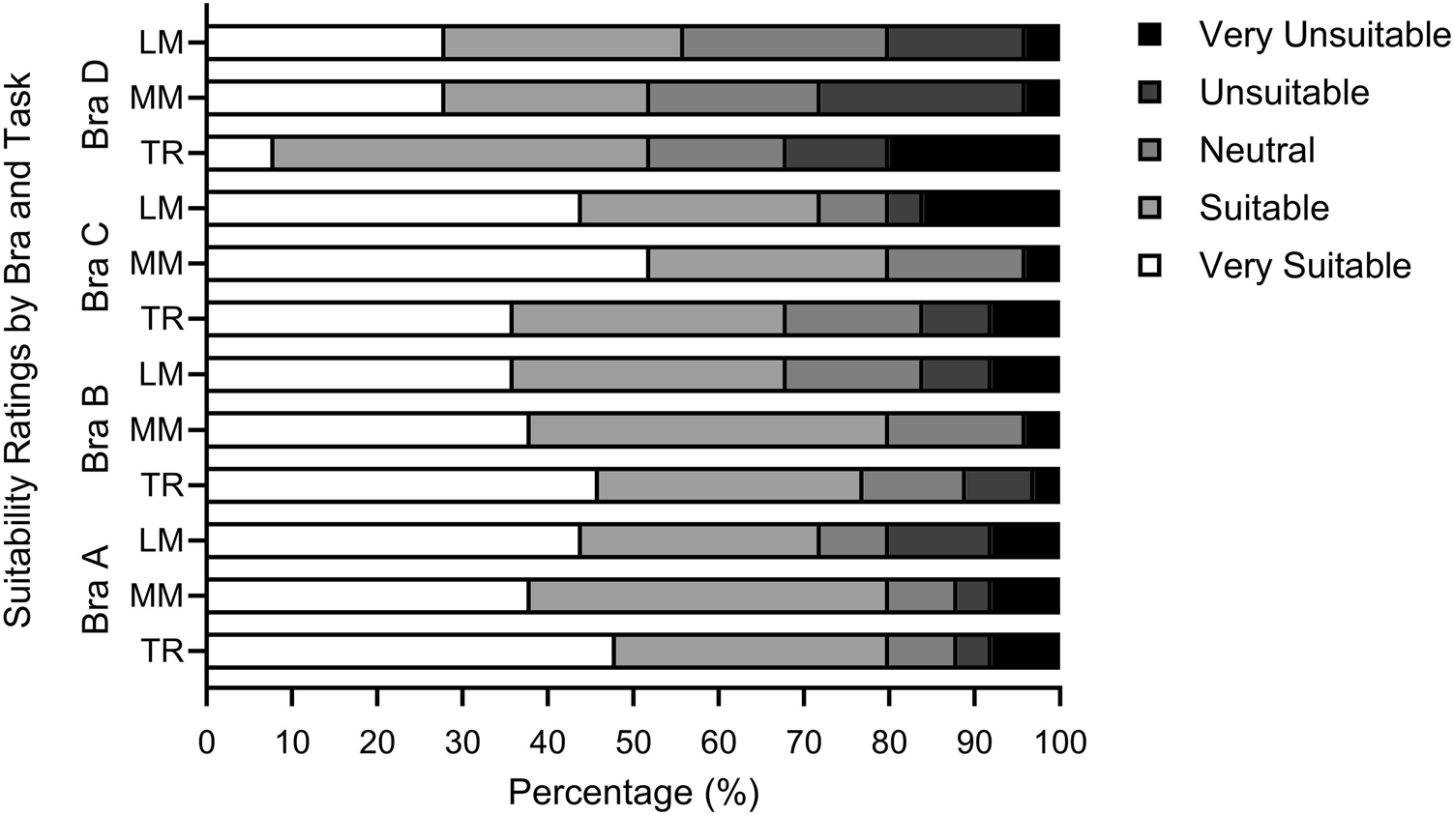
Percentage distribution of Likert Scale responses for suitability across tasks (loaded march [LM, military movements [MM] (foot drill, drop landing, burpee), treadmill running [TR]) in each bra condition (*n* = 25)

Across all bras and tasks, there were mixed responses from participants regarding their perceived suitability, with all bras being rated as both ‘very unsuitable’ and ‘very suitable’ for each task (Fig. 4), highlighting individual preferences. Bras A and B were more frequently rated as ‘very suitable’ for treadmill running, despite reported difficulties with donning and doffing, with Bra D rated as least suitable for this task. Bra C was considered the most suitable (52%) for a foot drill, suggesting that participants valued a combination of support and comfort. Detailed comments related to bra suitability are given in the ESM.

#### Thermal Considerations

In all four bras, a significant decrease in skin temperature pre to post the loaded march was observed (ESM). Bra D had the largest (mean ± standard deviation) reduction in skin temperature (− 1.58 ± 0.98 °C) [*t*_(24)_ = 7.888,* p* ≤ 0.001, *d* = 1.36] (ESM). There was a significant difference in delta skin temperature pre to post the loaded march between the four bras (*F*_(3)_ = 11.083,* p* ≤ 0.001). Delta breast skin temperature was found to be significantly greater when wearing bra D (− 1.58 ± 0.98 °C) compared with bra A (− 0.60 ± 0.78 °C) and bra C (− 0.65 ± 0.96 °C) [*t*_(23)_ = 4.447,* p* ≤ 0.001, *d* = 1.06; *t*_(23)_ = 4.666,* p* ≤ 0.001, *d* = 0.96 respectively]. Sports bra mass was significantly different across the four bras both pre (*f*_(3,72)_ = 46.146,* p* ≤ 0.001) and post (*f*_(3,72)_ = 60.979,* p* ≤ 0.001) the loaded march. The change in bra mass (mean ± standard deviation) from pre to post the loaded march is significantly larger in bra D (11.12 ± 5.65 g) [i.e. more sweat collected] than bras A (9.32 ± 5.50 g), B (9.56 ± 5.06 g) and C (7.32 ± 4.07 g) [*t*_(24)_ = 2.895,* p* = 0.008, *d* = 0.32; *t*_(24)_ = 3.674,* p* ≤ 0.001, *d* = 0.29; *t*_(24)_ = 5.447,* p* ≤ 0.001, *d* = 0.77 respectively]. There were no significant differences found in rating of perceived exertion (all* p* > 0.05, range* p* = 0.196 to* p* = 0.933), thermal sensation (all* p* > 0.05, range* p* = 0.135 to* p* = 0.758), thermal comfort (all* p* > 0.05, range* p* = 0.069 to* p* = 0.957) or skin wettedness (all* p* > 0.05, range* p* = 0.052 to* p* = 0.911) between bras at any timepoint during either bout of the loaded march.

#### Bra Ranking

**Fig. 5:**
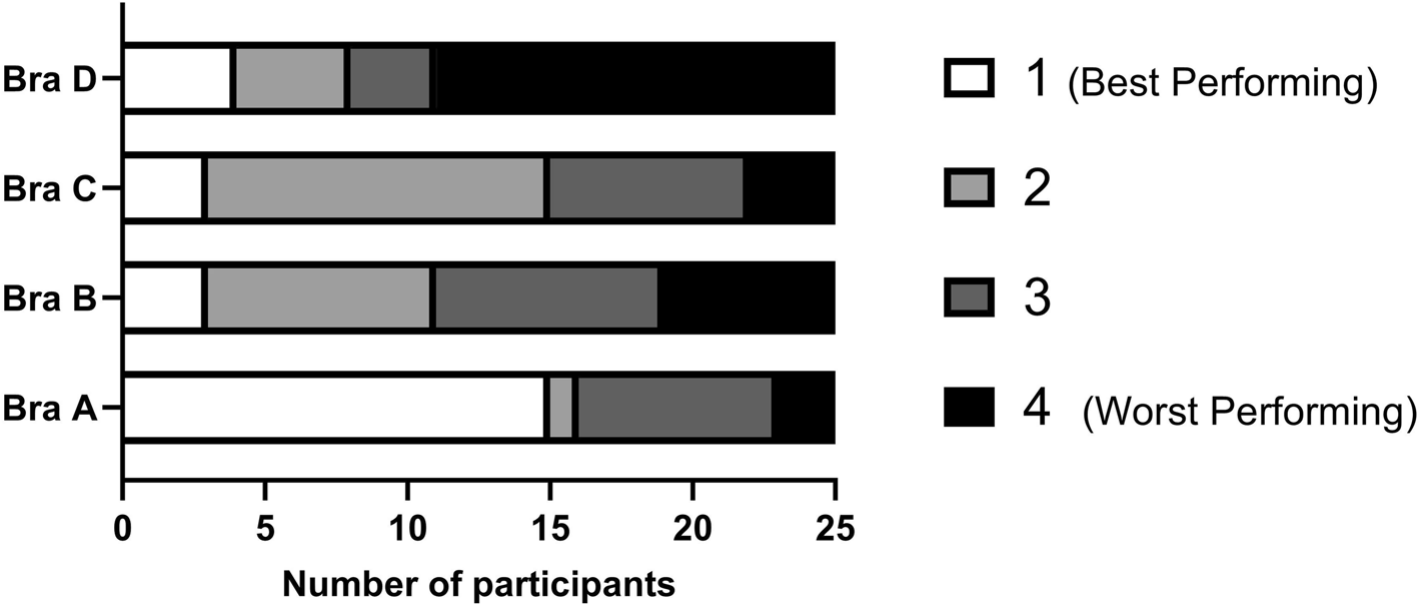
Overall ranking of the four sports bras in Study 2 from best to worst performing (*n* = 25)

After completing all simulated tasks, Bra A was ranked as the best performing and Bra D as the worst (Fig. 5). While Bras B and C received similar rankings, Bra B was rated worst slightly more often. However, variability existed, with 60% of participants ranking Bra A as the best, while 40% rated it as one of the worst, indicating potential reluctance to wear it for future tasks.

Despite its high ranking, Bra A was perceived to be the least comfortable for long-duration wear. In contrast, Bras C and D, which were often ranked lowest for performance, were considered more comfortable over extended periods. This suggests that while support is prioritised for short-duration activities, comfort becomes more important for prolonged wear.

## Study 3 (Long-Duration Use Field Experiment)

### Methods

#### Study Design and Participants

Study 3 adopted a longitudinal qualitative design using questionnaires and focus groups to explore participant’s experiences over time. Because of limited female recruitment into BT during 2023 (Ministry of Defence, 2023), the sample size was determined primarily by the time available for data collection rather than by statistical considerations. Over a 4-month period (February to May 2023), 103 female recruits were verbally briefed on the study, of whom 93 volunteered to participate (Table 1). Forty percent of participants were lost to follow-up because of discharges or re-squadding. Fifty-six participants completed the final study questionnaire. Nine focus groups (*n* = 33; 36%) were conducted, one with each study cohort.

#### Procedures

During the first week of BT, female participants attended the standard British Army BraFIS (delivered by the same contracted supplier as Study 1) to be fitted with the four bras tested in Study 2 (Sect. 2.3). Participants received an information sheet for each bra, detailing the tasks it was best suited for based on the findings from Study 2. Participants had the issued bras throughout BT, choosing how, when and for which tasks to wear them. They were asked to follow the bra information sheet for their first attempt at each task but could later swap between bras. If multiple bras were recommended for a task, the choice was theirs. The weekly questionnaire captured reasons for not wearing the issued bras during training (ESM).

Three questionnaires were used: an initial questionnaire to gather breast health history and participant demographics (completed in week 1 of BT) [ESM]; a weekly questionnaire to understand the bra-wearing habits of that week and identify any issues that arose with the bras (completed weeks 2–12) [ESM]; and a final questionnaire to assess the long-duration suitability of each of the bras (weeks 12 or 13) [ESM]. After the final questionnaire, semi-structured focus groups (3 or 4 participants) were held in the last 2 weeks of BT (ESM). Sessions were audio-recorded for later transcription.

#### Data Analysis

Descriptive analysis (e.g. frequencies and percentages) summarised demographics, bra support, comfort, fit, task suitability and wearing habits during BT. Free-text responses underwent a content analysis to identify key themes. Percentages were calculated based on the number of participants in BT during the corresponding week, with adjustments made to account for participant drop-outs.

Focus groups were transcribed verbatim, checked by the same researcher and analysed using an inductive content analysis [35]. Comment frequency for each theme and dimension was calculated and presented with example comments.

### Results

#### Questionnaires

Out of 56 respondents to the final questionnaire, 96.4% (*n* = 54) wore at least one of the issued bras during BT. Forty-seven percent (*n* = 26) wore the issued bras more than 5 days a week and 62.9% (*n* = 34) for more than 8 h per day. Overall, bra A was worn by the most participants (82%), closely followed by bra D (80%) and bra C (74%); bra B was worn by the lowest number of participants (59%). Throughout the BT period, there was a noticeable decline in the frequency of bra wear among participants. A common issue affecting usage was the unavailability of bras because of extended laundry timelines.

Participants reported issues with all bras during BT, particularly in the first 3 weeks when they were encouraged to trial each option. After which, there was a reduction in bra issues reported on a weekly basis, likely owing to participants choosing the bras that most suited their needs. The most common issue for bras A and B was ease of use; for bra C, it was fit and style; and for bra D, the material (ESM).

When asked to rate all four bras in terms of the support provided, participants rated bra C as ‘very supportive’ most often. Bra B was least often rated as ‘very supportive’ and had the highest percentage of participants (31%) reporting breast movement inside the bra, potentially because of poor fit or design. When asked to rate all bras in terms of their comfort across BT, bra D was most often rated as ‘very comfortable’, followed by bra C. Participants reported the highest incidents of rubbing and chafing whilst wearing bra A (34%), with the edge of the cups being the most frequent location for rubbing and chafing to occur.

Bra A was most frequently worn for a loaded march and outdoor PT, while bras C and D were preferred for classroom activities. Bra B was rated the least suitable overall, with bras A and C outperforming bra D in most tasks. During the initial weeks of BT, bra D was commonly worn for the foot drill, but in later weeks when foot drill frequency increased, participants chose to wear the more supportive bra B.

Bra B was most frequently reported as less comfortable while wearing additional equipment (ESM). The Bergen caused the most interaction issues, followed by the rifle and body armour. Some individuals reported discomfort from the top clasps of bras A and B; bra C interacted most with the rifle, likely owing to the anterior adjustment fastener on the shoulder strap, which some participants found caused breast-skin friction injuries.

**Fig. 6:**
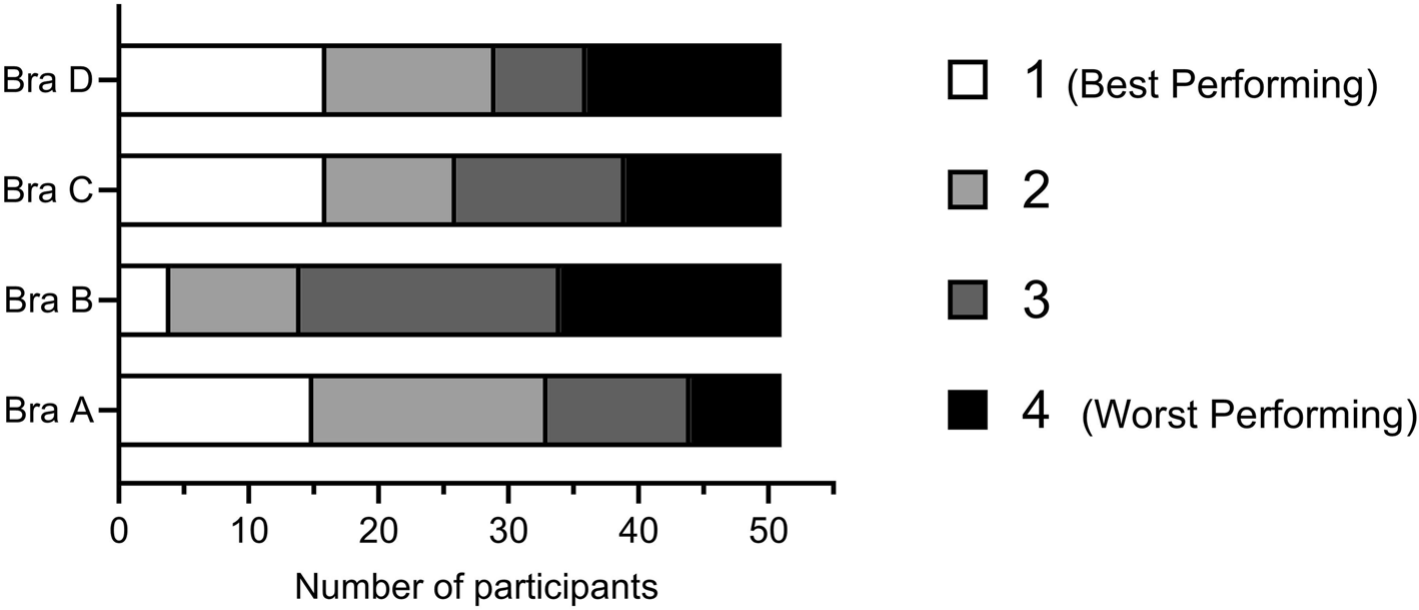
Ranking of the four bras from 1 (best performing) to 4 (worst performing) [*n* = 54]

Bras A, C and D were similarly ranked as best performing, it was clear that bra B was most often ranked as worst performing (Fig. 6). However, these data have a wide distribution, highlighting individuals’ preferences, experiences and, therefore, bra needs during BT.

#### Focus Groups

Data were categorised into nine higher order themes, with 32 general dimensions (ESM). Bra issues, BT tasks and the BraFIS were most frequently discussed (ESM); the fc and example comments for each bra are presented in the ESM. Rubbing and chafing was reported by 95% of focus group participants, with most incidents occurring in bras A and C during activities involving considerable arm movement (i.e. foot drills).

At the start of each focus group, participants were asked to identify their favourite or “best” sports bra for BT. Overall bra D (fc = 12), closely followed by A (fc = 10) and C (fc = 9), were most often identified as the “best” bras. Bra B was least often identified as the ‘best’ bra (fc = 2).

## Recommendations

Investigations of bra wear during short-duration simulated BT tasks (Study 2) and long-duration wear during BT (Study 3), following the evaluation of the current BraFIS (Study 1), have provided insights into the suitability of bras for BT. Recommendations of suitable sports bra characteristics for a range of different BT tasks have been produced (Table 3). Alongside recommended bra characteristics (Table 3), there were several additional recommendations resulting from recruit interviews and focus groups to improve the current BraFIS for female recruits in BT, some of which have already been implemented (Table 4).

**Table 3:** Overall bra characteristics recommended for basic training use

	PT sessions^d^	Field exercise^e^	Military tasks^f^	Foot drill	Classroom
Bra style	Combination/encapsulation	Compression	Combination/encapsulation	Any	Compression/encapsulation
Support level	High	Low/medium	High	High^b^	Low
Strap configuration	Racerback/cross back	Any^a^	Racerback/cross back^a^	Racerback/cross back	Any
Padding/moulded cups	Yes	Yes	Yes	Yes	Either
Neck drop	High	Medium	High	High	Any
Underband adjustability	Yes	Yes	Yes	Yes	Any
Strap adjustability	Yes	Yes	Yes	Yes	Any
Comfort	Important	Priority	Important	Priority^c^	Priority
Sizing	Underband and cup	Underband and cup	Underband and cup	Underband and cup	Underband and cup
Ease of use	Easy to don and doff, no high positioned top clasp				

*PT* physical training

^a^Any strap configuration but ensure no bulky clips or clasps

^b^Whilst recruits indicated this was low, movement seen during simulations suggests medium/high support for breast health (further research required)

^c^Lateral panels having no seams to prevent rubbing/chafing

^d^PT sessions are inclusive of gym cardio, gym strength and group sessions

^e^Field exercise relates to simulations of warfighting conditions in the field (i.e. outside), usually spanning multiple days

^f^Military tasks are inclusive of loaded marching, fire and movement, range activities and outdoor PT (obstacle course)

**Table 4:** Recommendations for improving the British Army sports bra fitting and issue service for BT

Recommendation	Details
Increase bra quantity	Three bras are insufficient for BT tasks. A minimum of five bras is recommended because of washing cycles
Modify laundry policy	Sports bras should not be tumble dried against manufacturer guidelines. They should be washed separately at a lower temperature and air dried or tumble dried on low heat if necessary
Individual fittings	Each bra should be fitted separately because of size variations between manufacturers
Immediate bra availability	Recruits should receive bras on the day of fitting to ensure proper breast support from the start of training and avoid administrative issues with incorrect sizes. If immediate provision is not possible, a clear exchange process should be in place
Second fitting during BT^a^	A second fitting is necessary to accommodate body and breast size changes during training
Availability in clothing stores	Bras should be stocked alongside other uniform items to simplify exchanges and replacements
Education programme	Introduce a bra fit and breast health education programme for recruits and training staff, covering fit, adjustment, care, hygiene and appropriate selection for BT tasks
Designated point of contact	Each BT site should have a designated contact to coordinate bra fittings in advance and ensure they occur in week 1 of training
Improve communication	Clearer communication is needed between quartermasters, training teams, bra fitters and recruits to prevent logistical issues (e.g. incorrect sizing or fitting delays)
Accurate kit list information	Recruits should receive consistent and clear guidance on the number and type of sports bras provided and whether they need to bring additional bras

*BT* basic training

^a^This was implemented by the British Army in 2024

## General Discussion

This research provides the first in-depth mixed-method evaluation of bra fit and function in female recruits during British Army BT, revealing a persistent yet reduced prevalence of bra-related issues. Reported problems decreased from 75% in 2021 to 61% (Study 1), a rate now lower than in other physically active populations, such as marathon runners (75%; [21]), although still high. This decline suggests that implementation of the BraFIS has begun to positively impact female recruits’ experiences. However, the persistence of issues, particularly related to fit, ease of use and integration with additional equipment, indicates that current provision still fails to meet all operational and physiological demands. These findings have significant implications for health, performance and retention, and reinforce the critical role of a female-specific kit in arduous environments. This rigorous programme of research delivers evidence-based recommendations that advance understanding of bra suitability for military tasks, informing future procurement and design decisions.

Recommending a single sports bra for BT, whether currently available on the market or issued to recruits, has limited long-term applicability, as specific models may become obsolete. Instead, defining key bra characteristics (Table 3), as done in previous research [15, 18], ensures the British Army’s BraFIS remains adaptable and sustainable. Understanding bra performance in this context is important for optimising support and function for tactical athletes.

A high level of breast support is essential for high-intensity activities such as running and jumping [15, 41, 42]. Study 2 identified PT sessions and some military tasks as particularly demanding on the breast, incorporating high-impact movements. Consequently, combination and encapsulation style bras, previously shown to offer superior support [17, 42, 43], are recommended for these tasks. The foot drill presented a unique case, with recruits reporting low perceived support needs despite observed high breast movement in laboratory simulations (Study 2). Given the frequency of a foot drill in BT, a high-support bra is advised for protection against breast pain and damage [22, 44, 45]. However, because of the prolonged close-range arm movements in this task, bras must also prioritise comfort by avoiding seams or bulky joins on the lateral panels to prevent chafing (Study 3).

A trade-off exists between breast support and comfort during military tasks, as components designed for adjustability often enhance support but may compromise comfort owing to interference with equipment [19]. During prolonged field exercises, recruits prioritised comfort over support because of extended wear. Given the comparatively lower support requirements, compression-style bras with no componentry, commonly worn by both general and recruit populations [11, 16], are recommended. Recruits rated bra D (compression) as the most comfortable for long-duration wear. However, the hygiene and material composition of a bra designed to be worn for multiple days should be considered.

The novel investigation of thermal properties in four sports bras during loaded marching (Study 2) also highlights the importance of material selection in sports bra design. Bra D was associated with the greatest reduction in breast skin temperature following the loaded march, suggesting enhanced cooling at the skin surface. However, it also showed the largest increase in bra mass, indicating greater sweat retention, which could reduce wearer comfort and increase chafing risk. This apparent contradiction may reflect a combination of higher sweat production (driven by thermoregulatory demands) and the material’s absorbency, which retains moisture despite surface cooling [46]. Unfortunately, because of laboratory constraints, it was not possible to reliably control or accurately monitor the thermal environment during testing, which may have influenced physiological responses. Future research should explore fabric properties that balance evaporative heat loss with effective moisture wicking, particularly considering regional sweating patterns across the breasts [47]. This is especially important for tactical athletes undertaking prolonged physical tasks in hot climates, both in the UK and abroad.

Key bra characteristics (Table 3), informed by recruit feedback, provide a basis for optimising sports bra procurement and guiding tactical athletes on task-specific bra selection. For example, padding or moulding within the cup is recommended for most tasks because of reported modesty concerns. Racerback or cross-back straps are preferred for security, but they should be free from bulky clips or clasps that could interfere with tactical equipment. Adjustable shoulder straps are essential for fit, and users should receive guidance on proper adjustment. Additionally, all bras should feature an adjustable underband to accommodate individual fit and changes throughout their lifespan. Adjustability components must be easily accessible, secure, and covered in soft or padded material to prevent skin damage. Bras sized by the underband and cup, rather than alpha sizing, are recommended for better fit. Finally, bras must be easy to don and doff independently, avoiding complex or hard-to-reach fastenings.

Military-specific tasks often require recruits to wear additional equipment (e.g. Bergen, webbing, rifle), which can impact bra comfort and functionality. Previous research has primarily focused on body armour integration, reporting issues such as discomfort, chafing, and breast injuries in military and police populations [48–50]. This is confirmed by Study 1 where recruits reported discomfort when wearing body armour (43%) and full-fighting order (45%), with all additional equipment reducing bra comfort regardless of style. Bras A and B were rated least comfortable in Study 3 because of a high-top clasp interfering with equipment. While such adjustment points are essential for fit, they should not interfere with equipment in the BT environment. While sports bras have been found to enhance comfort and support under body armour [51], they are not optimised for prolonged wear with ballistic vests [52], suggesting the need for the development of a tactical bra that meets conflicting demands. These findings are relevant to other tactical athlete populations, such as law enforcement and firefighters, highlighting the need for further research into equipment integration and the development of a purpose-designed tactical bra for prolonged wear.

Previous research has identified differences in breast and bra-related issues between small-breasted and large-breasted individuals [7, 9, 22]. However, Study 1 found minimal differences in recruits’ perceived breast-related task demands and no significant variation in overall bra-related issues between breast size groups during BT. As a result, subsequent studies did not differentiate between these groups, as the issues identified were broadly applicable to all recruits. Breast mass in Studies 1 and 3 was estimated using self-reported bra sizes, which are often inaccurate [37], and this is acknowledged as a limitation in participant grouping. The body mass index of the general population is however increasing [53] and given the established correlation between body mass index and breast size [20], female recruits may present with larger breasts in the future. To ensure inclusivity remains, a BraFIS must be able to provide bras across the full range of breast sizes, particularly those at the extremes.

This research has also led to key recommendations for improving the British Army’s BraFIS (Table 4). As a result of a policy change, recruits now receive an additional bra and a second fitting mid-course, addressing issues related to bra quantity and fit changes during BT. Integrating education alongside these changes could further enhance breast health and well-being [8, 54–56]. Educating recruits on fit and adjustment will help maintain optimal support despite washing and body composition changes, ultimately extending the lifespan of each bra.

The limitations of this programme of research were acknowledged as inherent challenges in study design and participant variability. For example, blinding participants to the bras used in Studies 2 and 3 was not possible, potentially introducing bias from preconceived perceptions of brands, despite efforts to emphasise that the research focused on bra characteristics. Additionally, while menstrual cycle phases can influence breast size and exercise-induced breast pain [57–61], exactly when or how these changes occur, or how they may affect breast movement and bra wear, alongside any effects of contraception is still unknown. Given that recruits must train regardless of the cycle phase, this factor was beyond the scope of this research. Although menstrual cycle stage may have influenced Study 2’s variables, a controlled approach was not possible because of logistical constraints, as requiring participants to attend at a specific cycle stage would have compromised study feasibility and recruitment.

Although the current BraFIS is specific to phase one BT, the outcomes of this research have informed similar initiatives across UK Defence services. As of November 2024, trained Army personnel are now entitled to a £50 annual allowance for sports bras [62]. Similarly, both the Royal Navy and Royal Air Force have introduced monetary allowances towards buying new sports bras for trained staff, with the Royal Navy also implementing a BraFIS to phase one recruits since March 2023 [63]. These policy changes highlight the impact of this initiative and the growing recognition of women’s health and well-being as essential to increasing female representation in demanding occupational roles. Yet, it is important that female tactical athletes receiving a monetary allowance for sports bras also have access to unbiased educational resources and bra recommendations to ensure the effective use of these funds.

## Conclusions

This novel programme of research is the first to systematically evaluate and address the breast support needs of female tactical athletes, combining laboratory and field studies to generate actionable evidence-based recommendations. Following implementation of the British Army’s BraFIS, bra-related issues decreased by 18%, and further improvements, such as issuing bras aligned with task-specific demands (Table 3) and refining the BraFIS process (Table 4), are expected to enhance comfort, reduce breast-related discomfort and support task performance. No single bra design was suitable for all tasks; thus, the British Army and similar organisations must provide a range of bras to accommodate varying task demands. Given the extended duration and intensity of bra wear in BT, far exceeding typical sports bra use, this research confirms the need for a tactical bra specifically designed for arduous occupational roles. The framework established here offers a transferable model to improve bra provision, breast health and occupational performance for female tactical athletes worldwide.

## Supplementary Information

Below is the link to the electronic supplementary material.

Supplementary file1 (PDF 1489 KB)

## Declarations

### Availability of data and material

Processed data and additional results are available in the ESM. The raw data generated in this study are the property of the Ministry of Defence and access may be granted upon an approved request through the corresponding author.

### Code availability

The code used in this study is available from the corresponding author on reasonable request.
