# Supplementary material for: Establishing Suitable Bra Characteristics for Tactical Athletes: A Mixed-Method Multi-Study Approach
Source: Sports Med. 2025 Dec 15;56(5):1255–71. doi: 10.1007/s40279-025-02375-3 (PMC13198511; doi:10.1007/s40279-025-02375-3)
Supplement: Supplementary file 1 — Supplementary file1 (PDF 1489 KB) [file 40279_2025_2375_MOESM1_ESM.pdf]

Title: **Establishing suitable bra characteristics for tactical athletes: A mixed-method multi-study approach**

Running Heading: **Suitable bra characteristics for tactical athletes: A mixed-method approach**

Authors: Emily Paines[1], Gemma Milligan[1], Mike Tipton[1], Andrew Roberts[2], Alex J Rawcliffe[2] & Jenny Burbage[1].

[1]*School of Psychology, Sport & Health Sciences, Research Group in Breast Health, University of Portsmouth, UK*

[2]*Army Recruit Health and Performance Research, Medical Branch, HQ Army Initial Training Command, Ministry of Defence, UK*

Corresponding Author: Dr Jenny Burbage jenny.burbage@port.ac.uk

ORCHID

Emily Paines (0009-0000-0928-7536)

Gemma Milligan (0000-0002-3539-915X)

Mike Tipton (0000-0002-7928-8451)

Andrew Roberts

Alex Rawcliffe (0000-0003-0942-7185)

Jenny Burbage (**0000-0002-7028-0381**)

Online resource 1. Characteristics of the four sports bras being tested.

Bra	Make	Model	Size range	Rank	% issued to recruits	Bra Style	Strap Adjustment	Strap configuration	Principal Fibre content	Underband adjustability / closure type / location	Cup padding	Neck Drop
A	Shock Absorber	Padded Run Bra	30-38<br>A-F	1	31.05%	Combination	Yes	Racerback	52% Nylon<br>33% Polyester<br>15% Elastane	Yes<br>Hook & eye<br>Back	Yes	Medium<br>(10.8 cm)
B	Shock Absorber	Ultimate Run Bra	30-38<br>A-G	2	18.18%	Combination	Yes	Racerback	81% Nylon<br>10% Polyester<br>9% Elastane	Yes<br>Hook & eye<br>Back	No	High<br>(9.8 cm)
C	Triumph	Traction Hybrid Lite Sports Bra	32-38<br>A-E	3	9.01%	Encapsulation	Yes	Straight straps	45% Nylon<br>37% Polyester<br>15% Elastane<br>3% Cotton	Yes<br>Hook & eye<br>Back	Yes	Low<br>(13.7 cm)
D	Sportjock	Action Sports Bra<br>Super Sports Bra*	XS-XL (A-C)<br>XS-XL (D-F)	5<br>12	7.49%<br>1.63%	Compression	No	Racerback	90% Polyester<br>10% Lycra	N/A	No	High<br>(8. 5 cm)

* Sportjock Action and Super are the same bra sold separately. To ensure the full-size range was available both were included as sports bra D.

Online resource 2 (Questionnaires, Focus Group and Interview Questions)

a) Study 1 Questionnaire

Section A: Personal Information
A.1. Service Number
A.2. Date of Birth
A.3. Training establishment
A.4. What is your most commonly worn everyday bra band size?
A.5. What is your most commonly worn everyday bra cup size?
A.6. Height (cm)
A.7. Weight (kg)
A.8. What is the highest degree or level of education you have completed?

Section B: Sports bra use and preferences
B.1. How often have you worn a sports bra since joining the Army?
B.2. How long (on average) do you wear your sports bra for (if applicable)?
B.3. Do you change your bra during the day?
B.4. What bra size were you issued with at the start of basic training?
B.5. What style of sports bra you were provided with at the start of BT?
B.6. Do you wear the sports bras which were provided to you at the start of basic training?
B.7. Why don't you wear the sports bras provided at the beginning of basic training?
B.8. What style of sports bra do you wear most often during basic training?
B.9. Have you had any problems with the sports bras provided to you at the start of BT (tick all that apply)?
B.10. If you could change anything to the sports bras provided to you what would it be?
B.11. How satisfied are you with the provided sports bras?
B.12. When in camp how regularly do you wash your sports bra (if applicable)?
B.13. How do you wash your sports bra (if applicable)?
B.14. How do you dry your sports bra (if applicable)?
B.15. Do you wear an everyday (fashion) bra underneath your sports bra (if applicable)?
B.16. Do you wear a second sports bra underneath your sports bra (if applicable)?

Section C: Basic training and sports bra use
C.1. What activities do you think challenge your fitness the most?
C.2. During which activities do you feel the most need to wear a sports bra?
C.3. To what extent do you feel that increased breast movement can negatively affect performance?
C.4. To what extent do you feel that breast pain can negatively affect performance?
C.5. To what extent do you feel that rubbing/chafing can negatively affect performance?
C.6. To what extent do you feel that bra straps digging in can negatively affect performance?
C.7. To what extent do you feel that underwire digging in can negatively affect performance?
C.8. Do you feel the sports bras issued are less comfortable to wear when worn in combination with additional equipment?

Section D: Breast history
D.1. Are you pregnant?
D.2. Have you given birth to a child/children?
D.3. Did you breast feed?

D.4. Have you ever undergone breast surgery? (e.g. breast augmentation, breast reduction, mastectomy etc)

b) Study 1 Recruit interview questions

Welcome/introduction
Overview of project
Reassurances e.g. there are no right or wrong answers, we are interested in all points of view.
If it is ok with you. All dialog will be recorded, this information will be made anonymous and will remain confidential.

Must have
What activities during Basic Training do you most feel the need for a sports bra and why?
Are there any specific activities or actions within these tasks which require more support or cause more issues and why? is it worse with different items of kit if so why?
Do you wear a different bra for different activities? If yes, what bra do you wear for which activity and why?
On a typical training day do you have time between sessions to change sports bras if needed?/when on exercise?
What does outdoor PT mean to you?

Nice to have
If you could change anything about the bras provided what would it be and why?
Do you notice the fit of the bras change throughout the course of training?
Are there any bras which you think would be better suited for the purpose of BT if so what and why?
How many sports bras do you think would be suitable for the purpose of BT?
Is there anything else that you feel is important for us as the research team to know?
Thank you

c) Study 1 Subject Matter Experts (SME) interview questions

Introduction questions:
1. Current location and job role?
2. How many years have you worked in this role?
3. What was your previous role?
4. Please confirm, of the statements below, which apply to you.
I have experience in:
i. A position of leadership where they have directed recruits to perform the task (e.g. PTIs) and have observed the task being performed.
ii. Experience in a position of leadership where they have directed recruits to undertake training (non-physical) and have observed training being performed.
iii. Witnessing the task being performed in an acceptable manner.
iv. Witnessing the task being performed unsuccessfully and can attest to the reasons for, and the consequences of, this failure.
v. Witnessing and/or performing the task using several techniques and can comment

on the advantages and disadvantages of these techniques.
vi. Delivering formal training on the task (e.g. teaching courses, developing training curricula, etc.).
5. Please provide a breakdown of Basic Training and the progression over 12 weeks.
6. Please can you provide a list of five tasks that you consider being the most physically demanding?

d) Study 2 Questionnaire

Section A: Participant information
A.1.Participant number
A.2.Bra condition
A.3. Bra size
A.4. Session

Section B: Treadmill running
B.1. Please rate the amount of pain (if any) you experienced?
B.2. If you did where on the breast did you experience this pain?
B.3. How supportive did this bra feel?
B.4. Which aspects of the bra do you feel were most supportive?
B.5. Which aspects of the bra do you feel were least supportive?
B.6. Did you feel your breasts moving inside the bra?
B.7. How comfortable did this bra feel?
B.8. Were there any aspects of the bra where rubbing or chafing occurred? (If so, where)
B.9. To what extent do you feel this bra is suitable for this activity?

Section C: Military movements
Foot drill
C.1. Please rate the amount of pain (if any) you experienced?
C.2. If you did where on the breast did you experience this pain?
C.3. How supportive did this bra feel?
C.4. Which aspects of the bra do you feel were most supportive?
C.5. Which aspects of the bra do you feel were least supportive?
C.6. Did you feel your breasts moving inside the bra?
C.7. How comfortable did this bra feel?
C.8. Were there any aspects of the bra where rubbing or chafing occurred? (If so, where)
C.9. To what extent do you feel this bra is suitable for this activity?
C.10. Did you notice any differences in support/comfort of the bra between different foot drills?
Drop landing
C.11. Please rate the amount of pain (if any) you experienced?
C.12. If you did where on the breast did you experience this pain?
C.13. How supportive did this bra feel?
C.14. Which aspects of the bra do you feel were most supportive?
C.15. Which aspects of the bra do you feel were least supportive?
C.16. Did you feel your breasts moving inside the bra?
C.17. How comfortable did this bra feel?
C.18. Were there any aspects of the bra where rubbing or chafing occurred? (If so, where)
C.19. To what extent do you feel this bra is suitable for this activity?

Burpee
C.20. Please rate the amount of pain (if any) you experienced?
C.21. If you did where on the breast did you experience this pain?
C.22. How supportive did this bra feel?
C.23. Which aspects of the bra do you feel were most supportive?
C.24. Which aspects of the bra do you feel were least supportive?
C.25. Did you feel your breasts moving inside the bra?
C.26. How comfortable did this bra feel?
C.27. Were there any aspects of the bra where rubbing or chafing occurred?
C.28. To what extent do you feel this bra is suitable for this activity?

Section D: Loaded marching
D.1. Please rate the amount of pain (if any) you experienced?
D.2. If you did where on the breast did you experience this pain?
D.3. How supportive did this bra feel?
D.4. Which aspects of the bra do you feel were most supportive?
D.5. Which aspects of the bra do you feel were least supportive?
D.6. Did you feel your breasts moving inside the bra?
D.7. Do you feel that the bra stayed in place during the trial?
D.8. How comfortable did this bra feel?
D.9. Do you feel that the comfort of the bra is affected by load carriage equipment?
D.10. Were there any aspects of the bra where rubbing or chafing occurred? (If so, where)
D.11. Do you feel that rubbing/chafing was made worse by the load carriage system?
D.12. To what extent do you feel this bra is suitable for this activity?
D.13. Are there any parts of the bra that caused issues during this task?
D.14. Did you notice any differences in the bra between the two load conditions?
D.15. Are there any ways this bra could be more suitable for load carriage?
D.16. On a scale of 0 to 10 how would you rate the overall performance of this bra in terms of breast pain, comfort, support and interaction with equipment.
D.17. How likely would you be to wear this bra for future load carriage tasks?

Section E: Treadmill running (no bra)
E.1. Please rate the amount of pain (if any) you experienced?
E.2. If you did where on the breast did you experience this pain?

Section F: Overall
F.1. Do you feel that this bra would be comfortable when worn for long periods of time?
F.2. What aspects of the bra did you like, if any?
F.3. Please describe why you liked these aspects.
F.4. Do you have any other comments about the bra?

e) Study 2 Ranking questionnaire

1.Participant number
2. Session
3. Please rank the four bras you have worn from best to worst for **treadmill running** where 1 is the bra you feel was best for that specific activity and 4 the bra which performed worst.
4. Please rank the four bras you have worn from best to worst for **Foot drill** where 1 is the bra you feel was best for that specific activity and 4 the bra which performed worst.

5. Please rank the four bras you have worn from best to worst for **Drop landing** where 1 is the bra you feel was best for that specific activity and 4 the bra which performed worst.
6. Please rank the four bras you have worn from best to worst for **Burpee** where 1 is the bra you feel was best for that specific activity and 4 the bra which performed worst.
7. Please rank the four bras you have worn from best to worst for **Loaded marching** where 1 is the bra you feel was best for that specific activity and 4 the bra which performed worst.
8. When thinking about **all the tasks** you have undertaken; please rank the four bras you have worn from best to worst where 1 is the bra you feel was best for that specific activity and 4 the bra which performed worst.
9.Are there any additional comments about any of the bras you wish to make?

f) Study 3: Breast Health History

Section A: Personal information
A.1. Date of Birth
A.2. Training establishment
A.3. Service Number
A.4. What is your current (or most commonly worn) everyday bra size (e.g. 34D)?
A.5. What is the highest degree or level of education you have completed?

Section B: Breast history
B.1. Are you pregnant?
B.2. Have you given birth to a child/children? (if no please skip to B.4)
B.3. Did you breast feed?
B.4. Have you ever undergone breast surgery? (e.g. breast augmentation, breast reduction, mastectomy etc)

g) Study 3: Weekly questionnaires

Section A: Information
A.1. Service Number
A.2. Did you wear ANY of the sports bras issued to you at the beginning of basic training?
A.3. Why didn't you wear any of the bras provided?

Section B: Bra A
B.1. Did you wear sports bra A this week?
B.2. Why didn't you wear sports bra A this week?
B.3. Did you wear bra A for the activities below? (If you did not do any of the activities this week, please select not relevant) (Activities: Loaded Marching; fire and movement; classroom; gym-strength; gym-cardio; gym-group sessions; Range; Foot drill; outdoor PT; Team sports)
B.4. Have you had any of the following problems with sports bra A

Section C: Bra B
C.1. Did you wear sports bra B this week?
C.2. Why didn't you wear sports bra B this week?
C.3. Did you wear bra B for the activities below? (If you did not do any of the activities this week, please select not relevant) (Activities: Loaded Marching; fire and movement;

classroom; gym-strength; gym-cardio; gym-group sessions; Range; Foot drill; outdoor PT; Team sports)
C.4. Have you had any of the following problems with sports bra B

Section D: Bra C
D.1. Did you wear sports bra C this week?
D.2. Why didn't you wear sports bra C this week?
D.3. Did you wear bra C for the activities below? (If you did not do any of the activities this week, please select not relevant) (Activities: Loaded Marching; fire and movement; classroom; gym-strength; gym-cardio; gym-group sessions; Range; Foot drill; outdoor PT; Team sports)
D.4. Have you had any of the following problems with sports bra C

Section E: Bra D
E.1. Did you wear sports bra D this week?
E.2. Why didn't you wear sports bra D this week?
E.3. Did you wear bra D for the activities below? (If you did not do any of the activities this week, please select not relevant) (Activities: Loaded Marching; fire and movement; classroom; gym-strength; gym-cardio; gym-group sessions; Range; Foot drill; outdoor PT; Team sports)
E.4. Have you had any of the following problems with sports bra D

Section F: Additional comments
F.1. Do you have any further comments about any of the sports bras you wore this week?

h) Study 3: Final questionnaire

Section A
A.1. Service Number
A.2. How often have you worn your issued sports bras during basic training?
A.3. Why didn't you wear any of the issued sports bras during training?
A.4. How long (on average) did you wear your sports bra for?
A.5. Did you change your sports bra during the day (e.g. for specific activities)?

Section B: Bra A
B.1. Did you wear sports bra A during basic training?
B.2.Why didn't you wear sports bra A?
B.3. How comfortable did this bra feel during basic training?
B.4. Were there any aspects of the bra where rubbing or chafing occurred?
B.5. How supportive did this bra feel?
B.6. Did you feel your breasts moving inside the bra?
B.7. How suitable was sports bra A for the following activities during basic training? (Activities: Loaded Marching; fire and movement; classroom; gym-strength; gym-cardio; gym-group sessions; Range; Foot drill; outdoor PT; Team sports)
B.8. Did you feel sports bra A was LESS comfortable to wear when worn in combination with additional equipment? (Body armour; daysack; bergen; webbing; rifle; Full fighting order)
B.9. How would you rate the overall performance of sports bra A in terms of breast comfort, fit, support and interaction with equipment (where applicable) during basic training?
B.10. Do you have any other comments about wearing sports bra A during basic training?

Section C: Bra B
C.1. Did you wear sports bra B during basic training?
C.2.Why didn't you wear sports bra B?
C.3. How comfortable did this bra feel during basic training?
C.4. Were there any aspects of the bra where rubbing or chafing occurred?
C.5. How supportive did this bra feel?
C.6. Did you feel your breasts moving inside the bra?
C.7. How suitable was sports bra B for the following activities during basic training? (Activities: Loaded Marching; fire and movement; classroom; gym-strength; gym-cardio; gym-group sessions; Range; Foot drill; outdoor PT; Team sports)
C.8. Did you feel sports bra B was LESS comfortable to wear when worn in combination with additional equipment? (Body armour; daysack; bergen; webbing; rifle; Full fighting order)
C.9. How would you rate the overall performance of sports bra B in terms of breast comfort, fit, support and interaction with equipment (where applicable) during basic training?
C.10. Do you have any other comments about wearing sports bra B during basic training?

Section D: Bra C
D.1. Did you wear sports bra C during basic training?
D.2.Why didn't you wear sports bra C?
D.3. How comfortable did this bra feel during basic training?
D.4. Were there any aspects of the bra where rubbing or chafing occurred?
D.5. How supportive did this bra feel?
D.6. Did you feel your breasts moving inside the bra?
D.7. How suitable was sports bra C for the following activities during basic training? (Activities: Loaded Marching; fire and movement; classroom; gym-strength; gym-cardio; gym-group sessions; Range; Foot drill; outdoor PT; Team sports)
D.8. Did you feel sports bra C was LESS comfortable to wear when worn in combination with additional equipment? (Body armour; daysack; bergen; webbing; rifle; Full fighting order)
D.9. How would you rate the overall performance of sports bra C in terms of breast comfort, fit, support and interaction with equipment (where applicable) during basic training?
D.10. Do you have any other comments about wearing sports bra C during basic training?

Section E: Bra D
E.1. Did you wear sports bra D during basic training?
E.2.Why didn't you wear sports bra D?
E.3. How comfortable did this bra feel during basic training?
E.4. Were there any aspects of the bra where rubbing or chafing occurred?
E.5. How supportive did this bra feel?
E.6. Did you feel your breasts moving inside the bra?
E.7. How suitable was sports bra D for the following activities during basic training? (Activities: Loaded Marching; fire and movement; classroom; gym-strength; gym-cardio; gym-group sessions; Range; Foot drill; outdoor PT; Team sports)
E.8. Did you feel sports bra D was LESS comfortable to wear when worn in combination with additional equipment? (Body armour; daysack; bergen; webbing; rifle; Full fighting order)
E.9. How would you rate the overall performance of sports bra D in terms of breast comfort, fit, support and interaction with equipment (where applicable) during basic training?
E.10. Do you have any other comments about wearing sports bra D during basic training?

Section F: Ranking

F.1. Reflecting on the whole of your basic training, please rank the four issued sports bras in terms of overall performance from 1(Best performing) to 4 (Worst performing).
F.2. Do you have any other comments about the current sports bra fit and issue service?

i) Study 3: Focus group

Welcome
• Introductions – Our role i.e. To facilitate discussion
• Project/study aim – To improve the current service, specifically to understand how these sports bras and characteristics perform across the long duration of BT.
• How the session will run – Semi-structured so discussions are encouraged, there will be questions about the bras and also the service as a whole
• Reassurances e.g. There are no right or wrong answers, we are interested in all points of view. Please feel free to share your opinions. All dialog will be recorded, this information will be made anonymous

Questions
1. What was your sports bra preference for Basic Training and why?
2. Did you wear different sports bras for different activities? If yes, did you follow the information provided on recommended use? If not, why not, how did this differ to the info sheet?
3. How useful was the information provided on recommended use of the four issued sports bras?
4. How suitable were the sports bras across the range of different activities you undertook during Basic Training?
5. Which sports bra characteristics (e.g. style, material, neckline, adjustability, padding) did you feel were important to performance during Basic Training? Did these differ by activity?
6. Did you suffer from any breast or bra issues during Basic Training as a result of the sports bras issued?
7. How did you find the bra fit and issue service? Do you have any recommendations for improvement?
8. How did you find the quantity of sports bras issued and the washing process?

Wash up
Bring together findings
Any questions/additional comments not already mentioned
Thank you

Online resource 3. Criteria for Subject Matter Experts (SMEs) adapted from Blacklock et al. (2015).

	Criteria
i	Experience in a position of leadership where they have instructed recruits to undertake physical training and have observed training being performed.
ii	Experience in a position of leadership where they have instructed recruits to undertake non-physical training and have observed training being performed.
iii	Have witnessed training being performed in a correct manner.
iv	Have witnessed training performed incorrectly and can give the reasons for, and the consequences of, this failure.
v	Experience witnessing and/or performing training using several techniques and can comment on the advantages and disadvantages of these techniques.
vi	Experience delivering formal training on the task (e.g. teaching courses, developing training curricula, etc.).

*These six criteria represent criteria numbers 5 to 9 for identifying Subject Matter Experts (SMEs) as presented by Blacklock et al. (2015). Criteria 1 to 4 were omitted from this research as the purpose of this task analysis was the training that took place not the actual performance of the tasks.

Online resource 4. Sports bra wear frequency, wear duration and wear habits of female recruits in Basic Training (BT) across breast size groups.

**Frequency**	**Breast size group**					
	**Small-breasted (n = 179)**		**Large-breasted (n = 54)**		**All (n = 233)**	
	**n**	**%**	**n**	**%**	**n**	**%**
Always (7 days a week)	75	41.9	27	50.0	102	43.8
Often (5-6 days a week)	58	32.4	18	33.3	76	32.6
Sometimes (3-4 days a week)	21	11.7	5	9.3	26	11.2
Rarely (1-2 days a week)	6	3.4	0	0.0	6	2.6
Specific training activities (e.g. PT sessions)	14	7.8	4	7.4	18	7.7
Never	5	2.8	0	0.0	5	2.1
**Duration**	**Small-breasted (n = 180)**		**Large-breasted (n = 53)**		**All (n = 233)**	
	**n**	**%**	**n**	**%**	**n**	**%**
All day	80	44.4	27	50.9	107	45.9
More than 8 hours	54	30	17	32.1	71	30.5
6-8 hours	15	8.3	3	5.7	18	7.7
4-6 hours	11	6.1	4	7.5	15	6.4
2-4 hours	8	4.4	1	1.9	9	3.9
Less than 2 hours	12	6.7	1	1.9	13	5.6
**Do you change your bra during the day?**	**Small-breasted (n = 180)**		**Large-breasted (n = 54)**		**All (n = 234)**	
	**n**	**%**	**n**	**%**	**n**	**%**
Yes	79	43.9	18	33.3	97	41.5
No	101	56.1	36	66.7	137	58.5
**How many times (per day)**	**Small-breasted (n = 75)**		**Large-breasted (n = 16)**		**All (n = 91)**	
	**n**	**%**	**n**	**%**	**n**	**%**
Once	32	42.7	5	31.3	37	40.7

Twice	41	54.7	10	62.5	51	56
Three	1	1.3	1	6.3	2	2.2
**How regularly do you wash your sports bra?**	**Small-breasted (n = 177)**		**Large-breasted (n = 54)**		**All (n = 231)**	
	**n**	**%**	**n**	**%**	**n**	**%**
Every wear	58	32.8	17	31.5	75	32.5
After 2 wears	89	50.3	18	33.3	107	46.3
After 3 or more wears	30	16.9	19	35.2*	49	21.2

* Significant difference between small- and large-breasted recruits ($p < 0.05$)

Online resource 5. Issues with the sports bras provided by the fitting and issue service. Data presented across breast size groups. Expressed as n (number of participants) and percentage of participants.

	Breast size group					
	Small-breasted (n = 103)		Large-breasted (n = 38)		All (n = 141)	
	n	%	n	%	n	%
Fit	18	17.5	6	15.8	24	17
Size	15	14.6	7	18.4	22	15.6
Number of bras	18	17.5	9	23.7	27	19.1
Wash durability	6	5.8	5	13.2	11	7.8
Quality	1	1	0	0	1	0.7
Level of support	10	9.7	2	5.3	12	8.5
Style	11	10.7	3	7.9	14	9.9
Comfort	28	27.7*	3	7.9	31	22
No issues	39	37.9	17	44.7	56	39.7

* Indicated where a significant association lies (p<0.05).

Online resource 6. Problems (with example comments) the sports bras caused (n = 91). The frequency of comments (fc) relating to each category is also presented.

Category	fc	Example comment
Quantity	20	'Need more of them. 3 isn't a lot for the amount of physical activity we do'
Size/fit	17	'It feels too small, so I would change the size'
Comfort	10	'Make them more comfortable as most of mine rub under my arms'
Style	13	'Style. Quite a lot of the sports bras offered can be hard to do up and often we need to clip each other's up'
Colour	10	'The colour, we were all issued colours other than black and I have been told off because of it'
Second fitting	8	'My breast size has changed dramatically through the course of training so I have had to stop wearing the issued bra's as they no longer fit well. A second fitting would be awesome'
Wash Durability	5	'That they could withstand high levels of washing on high heat. Mine have shrunk'
Service	4	'Just to have more time when trying on the bras to make sure I picked the best ones for my shape'
Support	3	'A couple of my issued bras have turned out to not be very supportive when they were advertised as a high impact'
Appearance	1	'To look nicer'

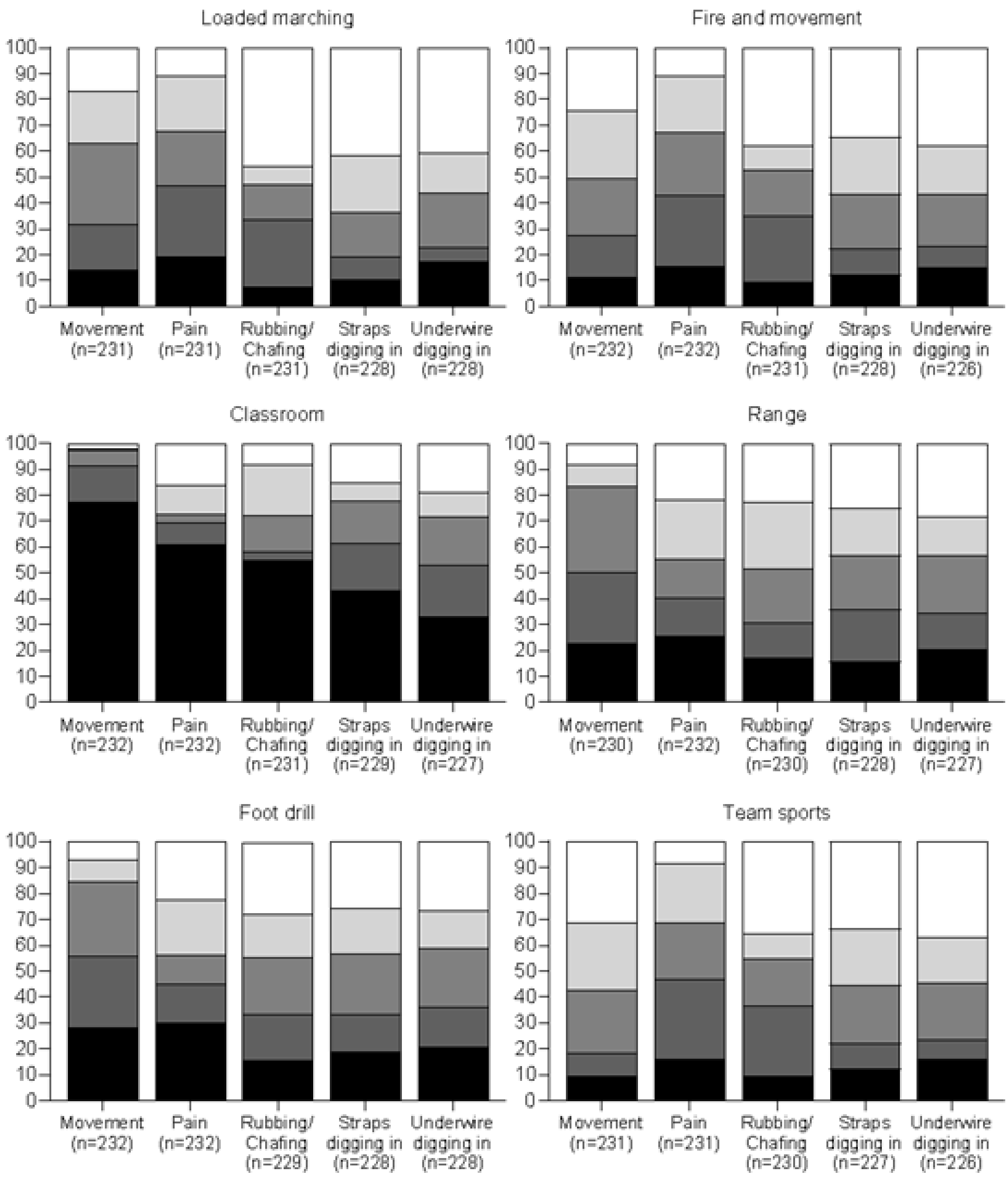
Loaded marching
Fire and movement
Classroom
Range
Foot drill
Team sports
0
10
20
30
40
50
60
70
80
90
100
Movement (n=231)
Pain (n=231)
Rubbing/ Chafing (n=231)
Straps digging in (n=228)
Underwire digging in (n=228)
Movement (n=232)
Pain (n=232)
Rubbing/ Chafing (n=231)
Straps digging in (n=228)
Underwire digging in (n=226)
Movement (n=232)
Pain (n=232)
Rubbing/ Chafing (n=231)
Straps digging in (n=229)
Underwire digging in (n=227)
Movement (n=230)
Pain (n=232)
Rubbing/ Chafing (n=230)
Straps digging in (n=228)
Underwire digging in (n=227)
Movement (n=232)
Pain (n=232)
Rubbing/ Chafing (n=229)
Straps digging in (n=228)
Underwire digging in (n=228)
Movement (n=231)
Pain (n=231)
Rubbing/ Chafing (n=230)
Straps digging in (n=227)
Underwire digging in (n=226)

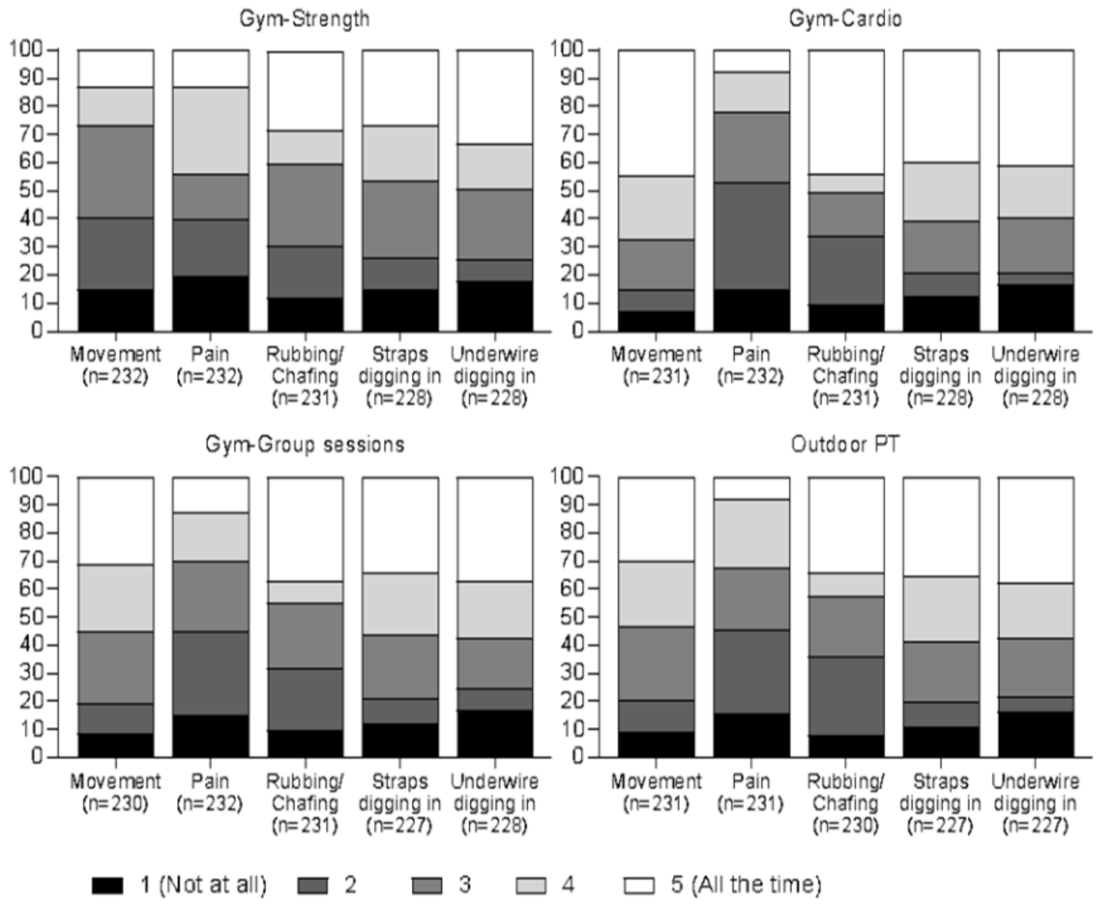

Online Resource 7. The extent to which participants perceived five breast or bra issues to negatively affect performance across ten Basic Training (BT) tasks. The y axes represent the percentage of participants; n represents the number of participants who responded to each question.

Online resource 8. Percentage of participants who indicated that bras were less comfortable when worn in combination with equipment across breast size groups. Expressed as n (number of participants) and percentage of participants.

	Breast size group					
	Small-breasted (n = 178)		Large-breasted (n = 54)		All (n = 232)	
	n	%	n	%	n	%
Body armour	72	40.4	27	50.0	99	42.7
Backpack	55	30.9	16	29.6	71	30.6
Webbing	74	41.6	21	38.9	95	40.9
Rifle	59	33.1	18	33.3	77	33.2
Full fighting order	79	44.6	24	44.4	103	44.6

Online resource 9. Themes identified from additional comments about the sports bra fitting and issue service identified by both recruits and SME (Subject Matter Expert) interviews, fc represents the frequency of comments and examples have been provided.

	Fc	Examples
Bra delivery time	5	Recruits<br>● Did not receive bras until week 14. When they did come they were not the same as ordered and were not suitable for a large-breasted recruit to wear, no contact information for returning or exchanging for a different style.<br>● Bras took 2-3 weeks to turn up.<br>SMEs<br>● Bras took 5-6 weeks to turn up leaving recruits without any sports bras for PT during the first few weeks of training.
Breast size changes during BT	4	Recruits<br>● Bras became too big through the course of training.<br>SMEs<br>● The bras are being measured tight because they expect recruits to lose weight but the bras are too tight to be used at the beginning of training – would be better to have a resizing session around week 8 to account for initial changes in body composition.
Access to bras	4	Recruits<br>● When the bra broke there was no way to replace them – need better access to bras throughout training rather than just during the initial fitting session.<br>● Had other appointment when the fitting was happening so never got given any bras at all – would have been good to have another opportunity rather than to miss out.
Number of bras – Confusion with Kit lists	6	Recruits<br>● Told to bring 5 (as this is what was needed for training) but only issued 3.<br>● Recruits told they were being provided with 10 but then only issued 3.<br>SMEs<br>● Recruits were told not to bring any bras as they were being provided – as these took 5-6 weeks to turn up recruits had no sports bras for PT.
Education	2	Recruits<br>● Would like to be educated on what bras were good for large-breast size and for different activities.<br>SMEs<br>● Suggested education programme for staff as well as recruits so that when recruits have questions staff are better informed to help recruits with any issues.
Other	1	Recruits<br>● Recruits felt like they wasted bras as they preferred their own and so did not use the ones provided.

Note: SME = Subject Matter Expert, BT = Basic Training, PT = Physical Training, Fc = Frequency of comments

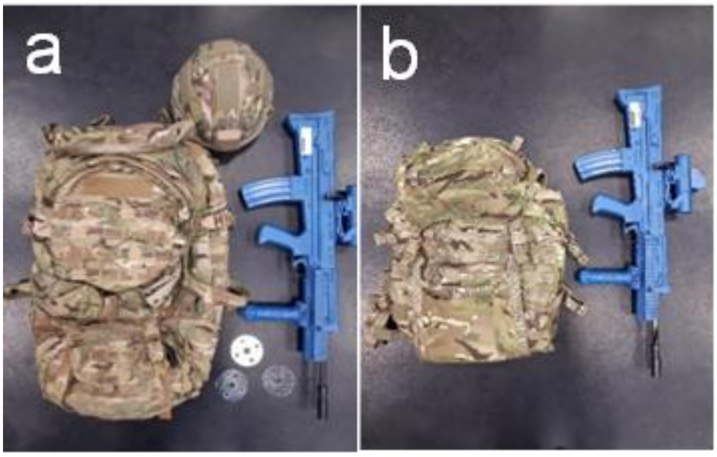

Online resource 10. A) loaded march equipment totalling 10 kg, as used in bout 1 of the load carriage task b) loaded march equipment totalling 7.5 kg, as used in bout 2 of the load carriage task.

Online resource 11. Summary of breast displacement statistical analysis in three directions and between the four bra conditions (Bras A, B, C, D).

		Vertical			Mediolateral			Anteroposterior		
		ROM	Peak positive	Peak negative	ROM	Peak positive	Peak negative	ROM	Peak positive	Peak negative
	F	45.877*	36.400	49.615*	28.292*	28.985*	17.538*	52.015*	44.077*	55.708*
	df	3	3	3	3	3	3	3	3	3
	p	<0.001	<0.001	<0.001	<0.001	<0.001	<0.001	<0.001	<0.001	<0.001
Post hoc (t-test)	A-B	0.075	0.038	0.02	0.291	0.075	0.515	<0.001	<0.001	<0.001
	A-C	<0.001	0.028	<0.001	0.635	1.000	0.423	0.009	0.018	0.012
	A-D	<0.001	<0.001	<0.001	<0.001	<0.001	<0.001	<0.001	<0.001	<0.001
	B-C	0.499	0.744	0.367	0.027	0.129	0.084	<0.001	0.005	<0.001
	B-D	<0.001	<0.001	<0.001	<0.001	<0.001	<0.001	<0.001	<0.001	<0.001
	C-D	<0.001	<0.001	<0.001	<0.001	<0.001	<0.001	<0.001	<0.001	<0.001

* Where data were non-parametric appropriate alternative tests were run

Note: ROM = Range of Motion

Online resource 12. Summary of breast velocity statistical analysis in three directions and between the four bra conditions (Bras A, B, C, D).

		Vertical		Mediolateral		Anteroposterior	
		Peak positive	Peak negative	Peak positive	Peak negative	Peak positive	Peak negative
	F	51.092*	42.969*	41.4*	55.975	40.338*	34.385*
	df	3	3	3	3	3	3
	p	<0.001	<0.001	<0.001	<0.001	<0.001	<0.001
Post hoc (t-test)	A-B	<0.001	0.009	0.041	0.013	0.165	0.38
	A-C	<0.001	<0.001	0.727	0.65	0.92	0.842
	A-D	<0.001	<0.001	<0.001	<0.001	<0.001	<0.001
	B-C	0.565	0.483	0.025	0.021	0.089	0.764
	B-D	<0.001	<0.001	<0.001	<0.001	<0.001	<0.001
	C-D	<0.001	0.004	<0.001	<0.001	<0.001	<0.001

* Where data were non-parametric appropriate alternative tests were run

Online resource 13. Summary of breast acceleration statistical analysis in three directions and between the four bra conditions (Bras A, B, C, D).

		Vertical		Mediolateral		Anteroposterior	
		Peak positive	Peak negative	Peak positive	Peak negative	Peak positive	Peak negative
	F	47.4*	40.8*	51.254	24.976	35.574	30.231*
	df	3	3	3	3	3	3
	p	<0.001	<0.001	<0.001	<0.001	<0.001	<0.001
Post hoc (t-test)	A-B	0.006	<0.001	0.006	0.125	<0.001	<0.001
	A-C	<0.001	<0.001	0.919	0.25	0.121	0.111
	A-D	<0.001	<0.001	<0.001	<0.001	<0.001	<0.001
	B-C	0.92	0.437	0.017	0.009	0.164	0.089
	B-D	<0.001	<0.001	<0.001	<0.001	<0.001	<0.001
	C-D	<0.001	<0.001	<0.001	<0.001	<0.001	<0.001

* Where data were parametric appropriate alternative tests were run

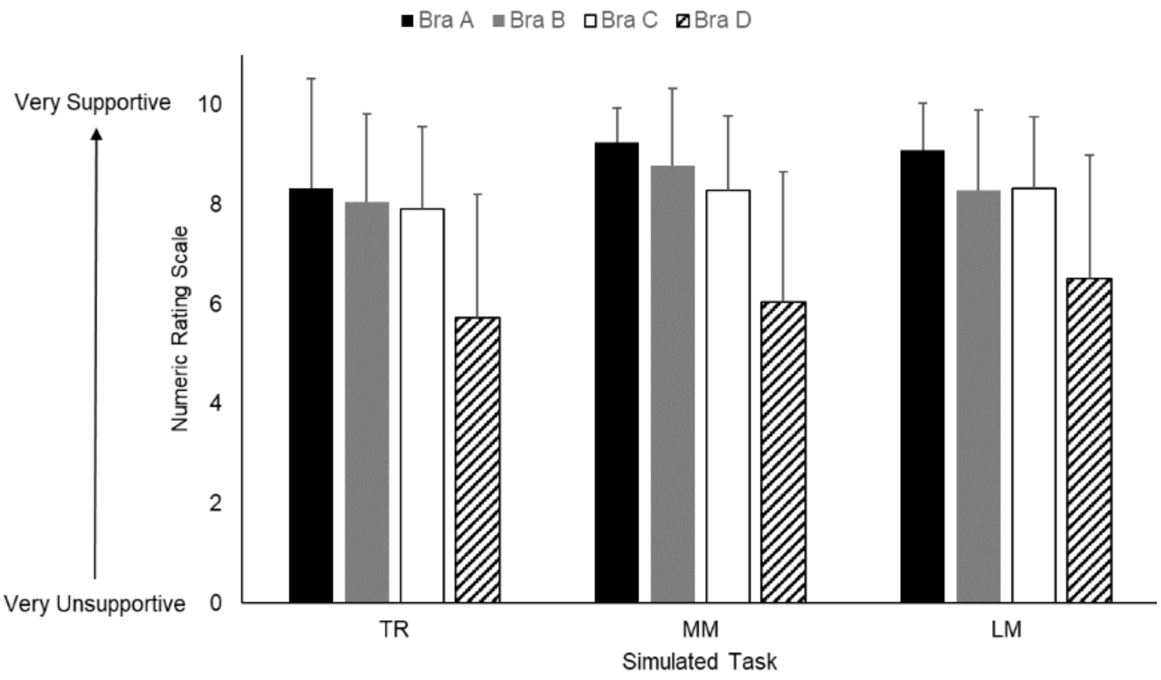

Online resource 14. Mean (SD) ratings of support across simulated laboratory tasks (LM: loaded march; MM: military movements [foot drill, drop landing, burpee]; TR: treadmill running) in each bra condition (n = 25).

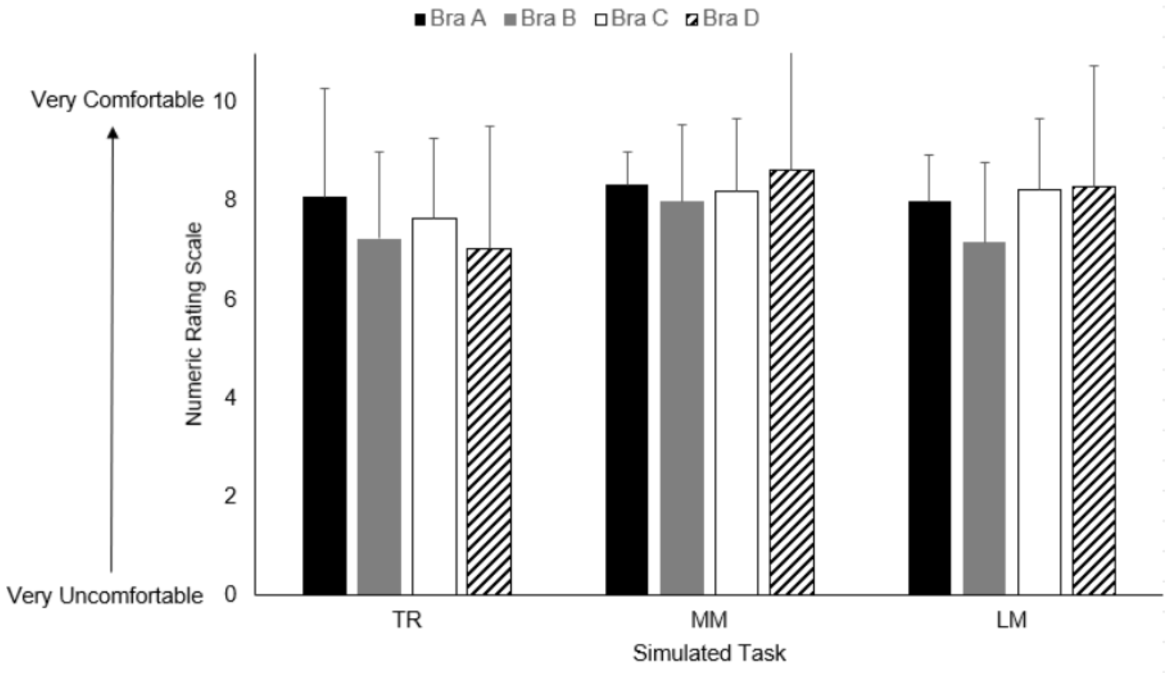

Online resource 15. Mean (SD) ratings of comfort across simulated laboratory tasks (LM: loaded march; MM: military movements [foot drill, drop landing, burpee]; TR: treadmill running) in each bra condition (n = 25).

Online resource 16. Why were the four sports bras suitable/unsuitable for the task of treadmill running. Questionnaire responses for bra A*.

Bra	Factor	Positive	Negative	Example comments
**A** 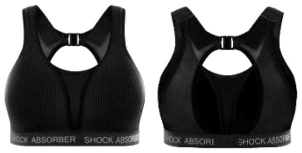	**Support**	18	1	Perfect support all round<br>The shoulder straps were sturdy<br>Mostly very supportive<br>Good support<br>Wasn't supportive enough for the speed and movement which comes with the speed
	**Comfort**	12	2	Comfortable and supportive but not restrictive<br>Comfortable and no pain and supportive<br>Comfy and supportive<br>Comfy and not too tight in any places but cup is tight enough for full support<br>Very comfy and supportive<br>Very comfortable and supportive could wear for a long time. Feels breathable too<br>The straps could be more comfortable as they are quite stiff and feel a bit restrictive<br>Well supported but not that comfortable
	**Pain**	3	0	Comfortable and no pain and supportive<br>Breasts did not move and did not hurt to run<br>I did not experience discomfort and the bra provided good support.
	**Size/Fit**	2	3	Good support in the straps and band but neckline could be higher<br>Underband rode up quickly under boobs but straps felt like they did most of the support work<br>It was supportive however a bit of chaffing did occur on the side of cup near armpit
	**Ease of use**	0	2	Also difficult to do top clasp and I needed help!<br>Again it is difficult to put on and get off

*This was a free text question where participants could write about more than one characteristic.

Online resource 16 continued. Why were the four sports bras suitable/unsuitable for the task of treadmill running. Questionnaire responses for bra B*.

Bra	Factor	Positive	Negative	Example comments
**B**	**Support**	13	3	Perfect support across all areas
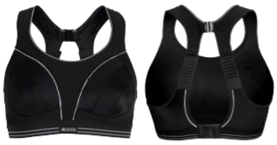				It supported my breasts and kept them in place
				Holds nicely in place, not much movement that I could feel
				Supportive
				No padding on cups so feels less supportive allowing more movement when running
				This one didn't feel as supportive. The cups weren't as tight and it felt more like a regular bra
				Comfortable to run, cups not as supported at high speed
	**Comfort**	7	3	Was comfortable when running, breasts didn't feel like they moved
				Very supportive and comfortable for this activity, no pain whatsoever
				Comfortably tight and supportive
				Very comfortable and supportive
				Comfortable to run, cups not as supported at high speed
				Very supportive but a little uncomfortable on the shoulder straps
				After some time wearing the straps have become uncomfortable but I feel like they couldn't be any looser otherwise I would feel unsupported
				Not comfortable enough. Under arms really dig in
	**Pain**	1	0	No pain whatsoever
	**Size/Fit**	1	2	Felt very supportive and didn't rub when running
				Under arms really dig in
				The bra cups are baggy
	**Ease of use**	0	2	It is difficult to do the bra up myself as the top clasp is out my reach!
				The bra itself is difficult to get on

*This was a free text question where participants could write about more than one characteristic.

Online resource 16 continued. Why were the four sports bras suitable/unsuitable for the task of treadmill running. Questionnaire responses for bra C*.

Bra	Factor	Positive	Negative	Example comments
**C**	**Support**	17	3	Supportive, fairly comfortable<br>No movement of breast tissue<br>The bra held my breasts down whilst I did the run and it was comfortable<br>Extremely supportive. Very conscious of restriction of breathing from underband<br>Very supportive<br>Supportive when running<br>The V cut leaves some room for movement<br>would probably be better for weightlifting than running<br>Without racer back the straps feel less supportive
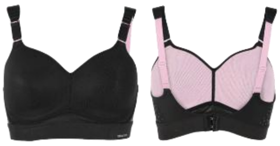				
	**Comfort**	8	1	Supportive, fairly comfortable<br>Was comfortable to run in, underband slightly rubbing but overall very good<br>It's fine for comfort, would probably be better for weightlifting than running<br>Very comfortable with good support<br>It's very supportive, comfortable, and feels durable<br>Comfortable, well supported<br>Good support but the shoulder straps were a bit uncomfortable
	**Pain**	1	0	It is very supportive in this high intensity exercise and I felt no pain, which I usually do
	**Size/Fit**	0	4	Did not cup my boobs<br>Less coverage of breast but meant it wasn't too warm<br>Not enough breast coverage
	**Ease of use**	0	0	

*This was a free text question where participants could write about more than one characteristic.

Online resource 16 continued. Why were the four sports bras suitable/unsuitable for the task of treadmill running. Questionnaire responses for bra D*.

Bra	Factor	Positive	Negative	Example comments
**D** 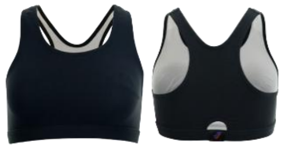	**Support**	11	8	Provides a reasonable amount of support in addition to being very comfortable<br>It is still very comfortable and supportive for fast running but a bit less so than the first one<br>Supportive and comfy but a little less than others<br>I felt supported and didn't feel my breasts moving<br>Very supportive for running<br>Too much breast movement, not supportive<br>This bra has no support!<br>It was comfy but not supportive, it felt like I was moving around a lot<br>Breast felt very unsupported when running, underband disappeared into my under boob and moved almost immediately<br>Over a longer period of time it wouldn't be supportive enough<br>Not as supported around the cups
	**Comfort**	8	2	very comfortable<br>It is still very comfortable<br>Supportive and comfy but a little less than others<br>It was comfy but not supportive, it felt like I was moving around a lot<br>Actually quite supportive, comfortable whilst running<br>It is comfortable and supportive without any chaffing<br>It's very comfortable while static but not as comfortable when running
	**Pain**	1	1	I did not experience any discomfort<br>Comfy to wear but when running breasts hurt as it was fairly unsupportive
	**Size/Fit**	2	0	Fabric felt breathable and underband held well<br>The shoulder straps and lateral panned maintained shape and fitted nicely
	**Ease of use**	1	0	It's easy to put on a it held my breasts in place

*This was a free text question where participants could write about more than one characteristic

Online resource 17. Why were the four bras suitable/unsuitable for the task of foot drill. Questionnaire responses for sports bra A*.

Bra	Factor	Positive (n)	Negative (n)	Example comments
**A** 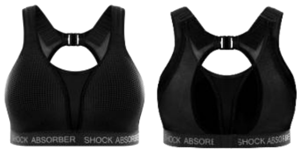	**Support**	22	0	Little movement - high support and comfort<br>No movement and full support, doesn't impact negatively at all<br>It held my breasts in place<br>Very supportive<br>Seemed to stop the majority of the bounce when marching and stamping<br>Well supported
	**Comfort**	11	2	Comfortable to wear, didn't feel breasts moving<br>Comfortable and secure<br>Very comfortable limited movement<br>Comfortable and soft, supportive<br>Supportive but not very comfy as the underband is too tight compared to the rest of the bra<br>Provides lots of support but is uncomfortable to wear due to large and inflexible panels (cups) and stiff straps
	**Pain**	0	0	
	**Size/Fit**	0	2	Support is super but it really rubs where shoulder straps rest on shoulder blades<br>Supportive back upper strap chafes after a while
	**Ease of use**	0	1	But difficult to do up with the clasps

*This was a free text question where participants could write about more than one characteristic.

Online resource 17 continued. Why were the four bras suitable/unsuitable for the task of foot drill. Questionnaire responses for bra B*.

Bra	Factor	Positive (n)	Negative (n)	Example comments
**B**	**Support**	21	2	Supportive but not restrictive
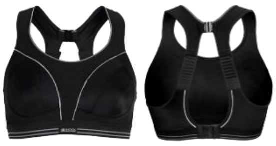				It held my breasts in place
				Very supportive and does not let my breasts move. No pain experienced
				Its suitable because it reduces movement
				No breast movement
				Lack of support in cup allows more movement especially during the stomp
	**Comfort**	11	1	Extremely comfortable very supportive
				Cups are comfortable and fabric soft
				Comfortable and supportive
				It was comfortable and caused no problems.
				Comfortable and no movement
				Comfortable and supportive
	**Pain**	1	0	No pain experienced.
	**Size/Fit**	0	0	
	**Ease of use**	0	0	

*This was a free text question where participants could write about more than one characteristic.

Online resource 17 continued. Why were the four bras suitable/unsuitable for the task of foot drill. Questionnaire responses for bra C*.

Bra	Factor	Positive (n)	Negative (n)	Example comments
**C** 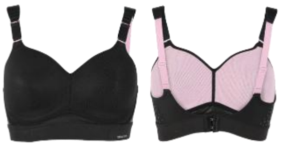	**Support**	17	0	Straps offer good support and underband feels secure<br>It held my breasts in place<br>Very supportive, comfortable and no breast pain.<br>No movement<br>Bra is very supportive on the shoulder straps and underband<br>Supportive all round when doing activities where the breasts bounce. Underband stayed in place throughout
	**Comfort**	11	2	Comfortable to wear and not too tight<br>Comfortable and supportive with no chafing<br>It is comfy enough to be worn for long periods of time while equally providing support<br>Comfortable, supportive. Only a little movement<br>Supportive just uncomfortable<br>Mostly comfortable. Only the straps are a bit uncomfortable
	**Pain**	1	0	No breast pain
	**Size/Fit**	1	3	No chafing<br>Too low cut and shoulder strap placement is uncomfortable and restrictive<br>Sometimes feels as if the straps will fall down
	**Ease of use**	0	0	

*This was a free text question where participants could write about more than one characteristic.

Online resource 17 continued. Why were the four bras suitable/unsuitable for the task of foot drill. Questionnaire responses for bra D*.

Bra	Factor	Positive (n)	Negative (n)	Example comments
**D**<br>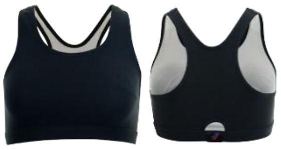	**Support**	6	13	Only slight movement during the stomp, the compression is enough so don't need padding on cups<br>Not much movement in the activity so didn't need a very supportive bra<br>Lots of movement of the breast<br>I could feel more movement compared to the other bras<br>No support in the bra<br>Comfortable when stationary however lots of breast movement<br>Breasts moving around even before stamping, breast movement is distracting<br>Not enough support
	**Comfort**	15	0	It is the most comfortable<br>It is very comfortable but use of no padding or adjustability there's lots of room for movement on the breast<br>Comfy but is not too tight<br>Very comfortable, good support<br>Comfortable fit but not particularly supportive
	**Pain**	0	0	
	**Size/Fit**	1	0	No rubbing when moving arms but not the most supportive
	**Ease of use**	0	0	

*This was a free text question where participants could write about more than one characteristic.

Online resource 18. Why were the four bras suitable/unsuitable for the task of drop landings. Questionnaire responses for bra A*.

Bra	Factor	Positive (n)	Negative (n)	Example comments
**A**	**Support**	20	2	Supportive during forceful landing
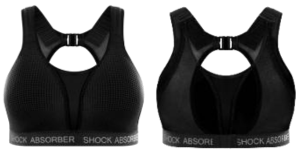				Full support all over
				Didn't feel like breast moved while doing drop landings
				It held my breasts in place
				Held breasts in nicely so didn't fall forwards from gravity (counter balance)
				Very supportive with no movement felt
				Lots of movement felt when landing
				Needs more supportive cup
	**Comfort**	5	1	High comfort and support
				Felt comfortable and didn't feel any movement
				Comfortable and supportive but difficult to get on with the clasps
				Comfortable and absorbs shock from landing
				not very comfy as the underband is too tight compared to the rest of the bra
	**Pain**	1	0	Supportive and I did not feel any pain
	**Size/Fit**	**0**	1	Shoulder straps rub wouldn't wear for long period
	**Ease of use**	**0**	2	The top clasp at the back is slightly too low so I needed help to do it up
				Comfortable and supportive but difficult to get on with the clasps

*This was a free text question where participants could write about more than one characteristic.

Online resource 18 continued. Why were the four bras suitable/unsuitable for the task of drop landings. Questionnaire responses for bra B*.

Bra	Factor	Positive (n)	Negative (n)	Example comments
**B**	**Support**	15	5	Tight and compress with no movement
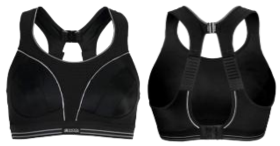				No breast movement
				It held my breasts in place
				Didn't feel breasts move much when landing
				Supportive and sturdy
				It was supportive enough felt really secure
				With the higher impact, the material around the ups wasn't supported enough
				Felt a lot of movement in the cup
	**Comfort**	1	0	It was comfortable and caused no problems
	**Pain**	1	1	Supportive and able to move without pain
				Lack of compression around breast from cup allows a lot of movement causing discomfort
	**Size/Fit**	**0**	1	Too much chaffing, I wouldn't want to wear this for long periods of time
	**Ease of use**	0	0	

*This was a free text question where participants could write about more than one characteristic.

Online resource 18 continued. Why were the four bras suitable/unsuitable for the task of drop landings. Questionnaire responses for bra C*.

Bra	Factor	Positive (n)	Negative (n)	Example comments
**C**	**Support**	16	4	Very supportive. Do feel like straps might slip off
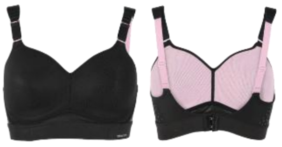				Under band and straps were highly supportive and comfortable
				It held my breasts in place
				I didn't notice any breast movement
				Less movement than other bras tried
				Overall kept breasts quite tight but did feel movement on the top where there was no support from bra
				It's slightly unsupportive and straps uncomfortable
	**Comfort**	8	1	Considering the force when you land, it was really comfortable
				Comfy good support
				Comfortable and supportive
				It is comfy enough to be worn for long periods of time while equally providing support
				It's slightly unsupportive and straps uncomfortable
	**Pain**	1	1	Supportive and no pain experienced
				Straps offer good support but the low cut allows movement of the top of the breast which causes some discomfort
	**Size/Fit**	2	0	Supportive without being uncomfortable or rubbing
	**Ease of use**	0	0	

*This was a free text question where participants could write about more than one characteristic.

Online resource 18 continued. Why were the four bras suitable/unsuitable for the task of drop landings. Questionnaire responses for bra D*.

Bra	Factor	Positive (n)	Negative (n)	Example comments
D	**Support**	6	14	Works for my small boobs but doubt it would for any bigger
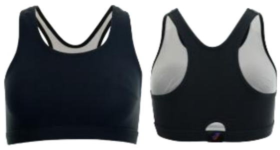				Comfy and I didn't notice breasts moving
				Bra has too much give allowing breast movement
				Breasts were moving quite a lot on landings
				Doesn't feel supportive
				No support
				It's not giving enough support
				Not enough support
				A fair amount of movement with the drop
				Not supportive enough for the landing
	**Comfort**	11	0	It's comfortable but I think the others are more supportive
				It was comfortable
				Comfortable for me whilst supportive and soft, also lightweight
				Very comfortable
				Good shoulder support and comfort
	**Pain**	1	0	no pain at all
	**Size/Fit**	0	0	
	**Ease of use**	0	0	

*This was a free text question where participants could write about more than one characteristic.

Online resource 19. Why were the four bras suitable/unsuitable for the burpee task. Questionnaire responses for bra A*.

Bra	Factor	Positive (n)	Negative (n)	Example comments
**A**	**Support**	13	2	Bra has slightly higher neckline so provides better support across the top of the breast
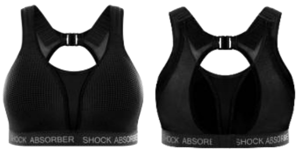				During all actions the support was high with great comfort
				Kept breasts in the same place and not too tight
				It held my breasts in place
				Little movement
				Provides good support
				Movement felt when jumping at the end of the burpee
	**Comfort**	8	2	Comfortable and no movement felt
				Comfortable
				Comfortable and supportive with no movement
				Comfortable but sometimes can feel breasts moving
				It’s uncomfortable (straps) and underband dug in when crouching
				The bra provides support but it’s not very comfy as the underband is too tight compared to the rest of the bra
	**Pain**	0	0	
	**Size/Fit**	0	2	Felt some rubbing afterwards but felt supportive
	**Ease of use**	0	1	The top clasp at the back is slightly too low so I needed help to do it up

*This was a free text question where participants could write about more than one characteristic.

Online resource 19 continued. Why were the four bras suitable/unsuitable for the burpee task. Questionnaire responses for bra B*.

Bra	Factor	Positive (n)	Negative (n)	Example comments
**B**	**Support**	14	3	No movement and tight to chest
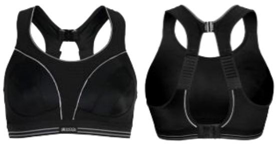				Minimal breast movement
				It held my breasts in place
				No bouncing or moving around when jumping
				Not much movement, at least not enough to feel discomfort
				Straps and underband provide good amount of support. If the neckline was slightly higher I feel this would provide more support in the cups
				*Comfortable but still some movement
				*More cup support needed
	**Comfort**	7	1	Back straps very comfortable and tight with nice material
				Supportive and comfy
				It was comfortable and caused no problems
				Comfortable but still some movement
				*uncomfortable
	**Pain**	1	1	My breasts were not moving, so I did not experience any pain
				*Lack of compression from cup allows more movement especially during jumping and kick back, noticeable discomfort
	**Size/Fit**	1	4	No chafing and very supportive
				*Shoulder straps felt too restrictive, underband flipped up when crouching down which was annoying and uncomfortable
				*It was supportive however chaffing did occur on sides of cups near armpit
				*Was supportive but side part of cup rubbed slightly when excessive arm moving
	**Ease of use**	0	0	

*This was a free text question where participants could write about more than one characteristic.

Online resource 19 continued. Why were the four bras suitable/unsuitable for the burpee task. Questionnaire responses for bra C*.

Bra	Factor	Positive (n)	Negative (n)	Example comments
**C** 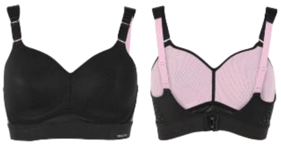	**Support**	12	3	Very supportive<br>Limited movement from breasts and comfortable to wear and move arms, wish there was material at the top of the bra to cover the whole breast<br>Felt secure enough<br>Bra is supportive and comfortable. With jump on the burpee the neck line could be higher just to provide a little more support at the front and top of the breasts<br>Underband stayed in place whilst moving around and bounce was minimal whilst jumping<br>Not enough stability in the cup, given its so low cut<br>Support isn't great and lack of coverage makes you feel really unsecure
	**Comfort**	6	1	Comfortable<br>It provided enough support required for the activity and was comfy<br>Bra is supportive and comfortable. With jump on the burpee the neck line could be higher just to provide a little more support at the front and top of the breasts<br>Very uncomfortable when lifting up the arms
	**Pain**	0	0	
	**Size/Fit**	1	2	No chafing when moving arms and breathable fabric<br>Felt some rubbing afterwards but felt supportive<br>It was supportive, however the lateral bands did rub a little
	**Ease of use**	0	0	

*This was a free text question where participants could write about more than one characteristic.

Online resource 19 continued. Why were the four bras suitable/unsuitable for the burpee task. Questionnaire responses for bra D*.

Bra	Factor	Positive (n)	Negative (n)	Example comments
**D**	**Support**	6	14	It was still comfortable and supportive enough but I would prefer a bra that makes my breasts move as little as possible
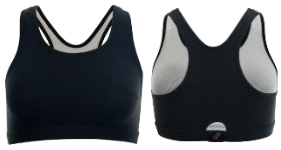				Very comfortable with minimal movement. Good Back support. Very smooth material
				No support
				Too much movement of the breast
				Too much movement in the breasts when jumping
				No support provided despite high comfort
				This bra feels like it gives no support
				Breast bouncing during jump is very distracting
				There was a bit too much movement during the jumping bits so the bra might not be that suitable for this activity
	**Comfort**	11	0	It was comfortable
				Comfortable but not as supportive
				Comfortable and not too much movement
				Felt comfortable
				Comfortable to stand still with
				Comfortable and supportive, did not slip
	**Pain**	0	0	
	**Size/Fit**	1	1	Comfortable and no chafing when arms moved but not a lot of support
				Support is lacking and shoulder straps rubbed at point arms were in the air on jump
	**Ease of use**	0	0	

*This was a free text question where participants could write about more than one characteristic.

Online resource 20. Why were the four sports bras suitable/unsuitable for the task of loaded marching. Questionnaire responses for bra A*.

Bra	Factor	Positive	Negative	Example comments
**A**	**Support**	17	0	Supportive but not restrictive
	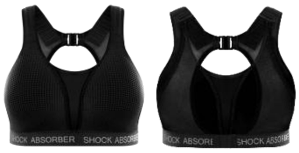			No movement of the bra, focused on the task rather than the bra
				Kept everything in place and allowed little if no movement. Felt supportive and comfortable for long periods of time
				Very supportive, good compression of the breasts
				It was very supportive but also very tight
				Suitable because it stayed in place and felt supportive. Didn't really notice it until I was hot
	**Comfort**	9	1	Comfortable and supportive
				Really comfortable did not feel the breasts moving at all
				Comfortable and didn't move and felt secure
				Comfortable no rubbing
				It was comfortable and supportive.
				Shoulder straps were uncomfortable and the longer it was worn the more uncomfortable it became
	**Pain**	0	0	
	**Size/Fit**	1	2	Comfortable no rubbing
				Really aggravated and chaffed around shoulder blades and straps especially where the adjustment straps are
				Unsuitable due to rubbing on shoulder blades, if wearing all day this may become worse
	**Ease of use**	0	0	

*This was a free text question where participants could write about more than one characteristic.

Online resource 20 continued. Why were the four sports bras suitable/unsuitable for the task of loaded marching. Questionnaire responses for bra B*.

Bra	Factor	Positive	Negative	Example comments
**B**	**Support**	15	2	Comfortable, enough support
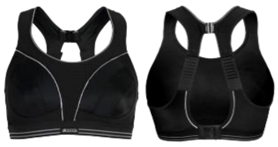				It was supportive
				Very supportive and didn’t interact with the load carriage equipment
				Provides okay support
				Very supportive
				Stayed in place throughout with minimal movement
				It does offer support, but could definitely be more supportive around the cup
				Does the job however due to no padding there is some movement which becomes uncomfortable
				Generally supportive, cup could have been more supportive
	**Comfort**	12	2	Comfortable and no breast movement
				Comfortable and supportive
				Comfortable and doesn’t slip, feels like you are concentrating on the activity and not distracted by how the bra feels
				Very comfortable and supportive, no chaffing
				It’s fine, it’s just not the most comfortable, supportive, or nice fabric
	**Pain**	0	0	
	**Size/Fit**	4	0	Held in place securely, didn’t rub too much
				Comfy and remained in place, no chaffing or discomfort even with the load
				Very comfortable and supportive, no chaffing
	**Ease of use**	0	1	It was supportive but the top clip opened

*This was a free text question where participants could write about more than one characteristic.

Online resource 20 continued. Why were the four sports bras suitable/unsuitable for the task of loaded marching. Questionnaire responses for bra C*.

Bra	Factor	Positive	Negative	Example comments
**C**	**Support**	20	0	It's supportive and was not restricting movement<br>Supportive but the underband was too tight<br>It's tight and supportive<br>Adequate support for larger-breasts<br>Very supportive and comfortable<br>Very secure<br>The bra was supportive however the cups did not fully fit the shape of my breasts.
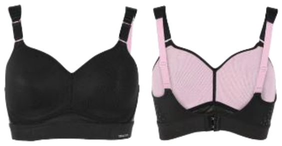				
	**Comfort**	13	1	Comfortable and supportive. Good padded cups<br>Comfortable and didn't move<br>It was comfortable and supportive<br>Comfy and supportive and straps didn't fall down although I was concerned that they might<br>Very comfortable, well supported throughout<br>Supportive and comfy<br>It's unsuitable, due to shoulder strap discomfort and placement
	**Pain**	0	0	
	**Size/Fit**	1	0	The load carriage did not cause rubbing and discomfort on the shoulder straps which could sometimes occur
	**Ease of use**	1	0	Fits well, is easy to put on and is supportive

*This was a free text question where participants could write about more than one characteristic.

Online resource 20 continued. Why were the four sports bras suitable/unsuitable for the task of loaded marching. Questionnaire responses for bra D*.

Bra	Factor	Positive	Negative	Example comments
**D**	**Support**	7	8	Little to no movement of breast at all, very comfortable<br>I don’t think the bra is unsuitable but it didn’t feel as supportive as the other bras<br>It was comfortable and smooth material. It was supportive with no chaffing<br>The bra is supportive enough for this exercise. The cup is not hard so doesn’t affect the bra comfort during load carriage
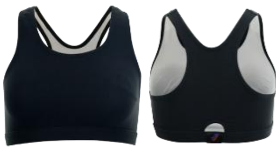				Very little support, very thin material, underband rolled and didn’t stay in place<br>It’s comfortable but doesn’t feel particularly supportive<br>Stayed in place but breasts didn’t feel completely supported
	**Comfort**	11	0	Comfortable but not completely supportive<br>Little to no movement of breast at all, very comfortable<br>Comfortable and very supportive<br>Suitable for comfort but maybe not support
	**Pain**	0	1	Would be suitable however it would cause pain in the future on the shoulders due to how tight it is
	**Size/Fit**	1	0	It was supportive with no chaffing
	**Ease of use**	0	0	
	**Thermal**	0	3	Thick fabric made breasts very hot, maybe suitable for winter wear<br>Material is not friendly for sweating and increases thermal sensation during marching<br>The cotton material feels very thick and hot

**This was a free text question where participants could write about more than one characteristic.

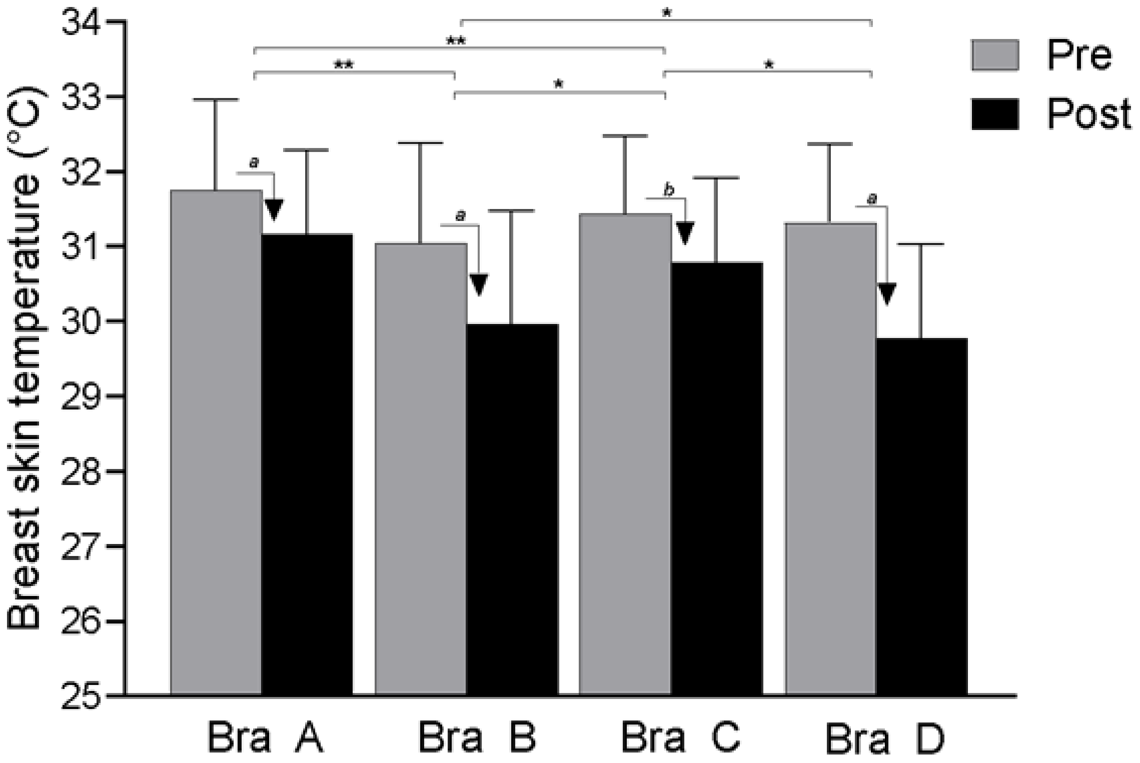

a. Significant change in temperature pre to post loaded march p ≤ 0.05.

b. Significant change in temperature pre to post loaded march p < 0.001.

*Significant difference in change in temperature between bra conditions p ≤ 0.05.

** Significant difference in change in temperature between bra conditions p < 0.001.

Online resource 21. Mean (SD) breast skin temperature (°C) of participants (n = 24) left breast pre and post loaded march task.

Online Resource 22. Reasons for not wearing issued bras across the weeks of Basic Training. n represents the number of participants that did not wear the bra that week, participants could select more than one reason.

	Week	n	In the wash	Style	Uncomfortable	Unsupportive	Wrong size	Prefer my own	Other
Bra A	2	17	0	0	1	0	0	1	15
	3	9	3	1	1	0	1	1	2
	4	14	2	2	2	0	1	3	5
	5	20	6	2	2	0	1	1	6
	6	13	2	2	3	0	1	1	3
	7	11	2	1	1	0	1	3	3
	8	11	2	2	1	0	1	3	2
	9	11	4	0	1	0	0	4	2
	10	10	3	0	1	0	0	2	4
	11	18	8	0	0	1	1	5	3
	12	10	5	0	1	0	1	2	1
**TOTAL**			**37**	**10**	**14**	**1**	**8**	**26**	**46**
Bra B	2	30	6	1	0	0	1	1	21
	3	25	5	4	1	0	1	6	7
	4	25	5	6	1	3	1	3	5
	5	29	6	3	0	3	1	4	10
	6	26	6	5	1	4	0	4	6
	7	23	4	5	0	1	0	5	9
	8	27	5	6	1	4	0	9	6
	9	13	1	0	1	2	1	3	6
	10	16	3	2	2	1	0	3	6
	11	20	4	3	3	5	1	5	2
	12	11	3	2	2	3	1	2	3
**TOTAL**			**48**	**37**	**12**	**26**	**7**	**45**	**81**
Bra C	2	16	3	1	1	0	0	1	10
	3	15	3	1	1	0	1	4	6
	4	15	3	2	1	1	0	2	7
	5	20	4	1	0	1	1	3	8
	6	20	4	2	0	0	2	3	6
	7	18	3	2	2	2	2	1	7
	8	16	7	1	1	1	3	1	2
	9	10	3	1	1	1	1	2	3
	10	7	0	0	1	0	1	1	3
	11	12	4	1	1	2	1	1	1
	12	9	3	0	2	1	1	1	2
**TOTAL**			**37**	**12**	**11**	**9**	**13**	**20**	**55**
Bra D	2	20	3	2	0	3	0	3	10
	3	14	3	1	2	3	0	2	3
	4	14	4	3	0	4	0	0	3
	5	18	7	2	0	0	0	3	6
	6	11	1	4	2	1	0	1	0
	7	13	2	1	1	1	0	2	5
	8	14	5	2	0	1	0	2	4
	9	12	3	3	1	0	0	2	3
	10	12	3	1	2	0	0	1	5
	11	8	2	1	0	2	0	1	2
	12	9	2	1	0	0	0	3	3
**TOTAL**			**35**	**21**	**8**	**15**	**0**	**20**	**44**

Online resource 23. Number of issues reported by participants wearing **bra A** across the weekly questionnaires. n represents the number of participants who both wore the bra that week and the number of participants who reported each issue. Participants could select more than one issue.

**Week**	**2**		**3**		**4**		**5**		**6**		**7**		**8**		**9**		**10**		**11**		**12**	
n who wore the bra this week	40		42		29		21		26		20		24		10		13		11		7	
	n	%	n	%	n	%	n	%	n	%	n	%	n	%	n	%	n	%	n	%	n	%
Fit	7	17.5	5	11.9	1	3.4	1	4.8	1	3.8	2	10.0	0	0.0	0	0.0	1	7.7	1	9.1	1	14.3
Ease of use	7	17.5	3	7.1	3	10.3	2	9.5	1	3.8	2	10.0	0	0.0	0	0.0	0	0.0	1	9.1	1	14.3
Size	6	15.0	3	7.1	2	6.9	1	4.8	1	3.8	2	10.0	0	0.0	0	0.0	1	7.7	1	9.1	1	14.3
Material	3	7.5	3	7.1	1	3.4	1	4.8	1	3.8	2	10.0	0	0.0	0	0.0	1	7.7	1	9.1	1	14.3
Wash durability	4	10.0	2	4.8	1	3.4	1	4.8	2	7.7	2	10.0	0	0.0	0	0.0	0	0.0	1	9.1	1	14.3
Style	3	7.5	2	4.8	1	3.4	1	4.8	1	3.8	2	10.0	0	0.0	0	0.0	1	7.7	1	9.1	1	14.3
Quality	4	10.0	2	4.8	1	3.4	1	4.8	1	3.8	2	10.0	0	0.0	0	0.0	0	0.0	1	9.1	1	14.3
Other	1	2.5	2	4.8	0	0.0	0	0.0	1	3.8	0	0.0	0	0.0	0	0.0	0	0.0	0	0.0	0	0.0

**The issues are ordered (top to bottom) from most frequently to least frequently identified across the duration of BT.*

Online resource 23 continued. Number of issues reported by participants wearing **bra B** across the weekly questionnaires. n represents the number of participants who both wore the bra that week and the number of participants who reported each issue. Participants could select more than one issue.

**Week**	**2**		**3**		**4**		**5**		**6**		**7**		**8**		**9**		**10**		**11**		**12**	
n who wore the bra this week	26		25		18		12		12		8		8		8		7		9		6	
	n	%	n	%	n	%	n	%	n	%	n	%	n	%	n	%	n	%	n	%	n	%
Ease of use	4	15.4	4	16.0	0	0.0	1	8.3	0	0.0	0	0.0	0	0.0	1	12.5	0	0.0	1	11.1	1	16.7
Fit	4	15.4	2	8.0	0	0.0	1	8.3	1	8.3	0	0.0	0	0.0	0	0.0	1	14.3	1	11.1	1	16.7
Size	4	15.4	2	8.0	0	0.0	1	8.3	1	8.3	0	0.0	0	0.0	0	0.0	1	14.3	1	11.1	1	16.7
Material	3	11.5	3	12.0	0	0.0	1	8.3	0	0.0	0	0.0	0	0.0	1	12.5	0	0.0	1	11.1	1	16.7
Wash durability	3	11.5	2	8.0	0	0.0	1	8.3	1	8.3	1	12.5	0	0.0	0	0.0	0	0.0	1	11.1	1	16.7
Style	4	15.4	2	8.0	0	0.0	1	8.3	0	0.0	0	0.0	0	0.0	0	0.0	0	0.0	1	11.1	1	16.7
Quality	3	11.5	2	8.0	0	0.0	1	8.3	0	0.0	0	0.0	0	0.0	0	0.0	0	0.0	1	11.1	1	16.7
Other	0	0.0	0	0.0	0	0.0	0	0.0	0	0.0	0	0.0	0	0.0	0	0.0	0	0.0	1	11.1	0	0.0

**The issues are ordered (top to bottom) from most frequently to least frequently identified across the duration of BT.*

Online resource 23 continued. Number of issues reported by participants wearing **bra C** across the weekly questionnaires. n represents the number of participants who both wore the bra that week and the number of participants who reported each issue. Participants could select more than one issue.

**Week**	**2**		**3**		**4**		**5**		**6**		**7**		**8**		**9**		**10**		**11**		**12**	
n who wore the bra this week	40		35		28		21		18		13		19		11		16		17		8	
	n	%	n	%	n	%	n	%	n	%	n	%	n	%	n	%	n	%	n	%	n	%
Fit	2	5.0	3	8.6	1	3.6	3	14.3	1	5.6	2	15.4	0	0.0	0	0.0	0	0.0	1	5.9	0	0.0
Style	3	7.5	3	8.6	2	7.1	2	9.5	1	5.6	1	7.7	0	0.0	0	0.0	0	0.0	1	5.9	0	0.0
Size	2	5.0	2	5.7	1	3.6	2	9.5	1	5.6	1	7.7	0	0.0	0	0.0	0	0.0	1	5.9	0	0.0
Quality	3	7.5	1	2.9	1	3.6	2	9.5	1	5.6	1	7.7	0	0.0	0	0.0	0	0.0	1	5.9	0	0.0
Ease of use	3	7.5	1	2.9	0	0.0	2	9.5	1	5.6	1	7.7	0	0.0	0	0.0	1	6.3	1	5.9	0	0.0
Material	2	5.0	1	2.9	1	3.6	2	9.5	1	5.6	1	7.7	0	0.0	0	0.0	0	0.0	1	5.9	0	0.0
Wash durability	2	5.0	1	2.9	1	3.6	2	9.5	1	5.6	1	7.7	0	0.0	0	0.0	0	0.0	1	5.9	0	0.0
Other	1	2.5	2	5.7	0	0.0	0	0.0	0	0.0	0	0.0	0	0.0	0	0.0	0	0.0	0	0.0	0	0.0

**The issues are ordered (top to bottom) from most frequently to least frequently identified across the duration of BT.*

Online resource 23 continued. Number of issues reported by participants wearing **bra D** across the weekly questionnaires. n represents the number of participants who both wore the bra that week and the number of participants who reported each issue. Participants could select more than one issue.

**Week**	**2**		**3**		**4**		**5**		**6**		**7**		**8**		**9**		**10**		**11**		**12**	
n who wore the bra this week	36		36		29		23		27		18		21		9		11		21		8	
	n	%	n	%	n	%	n	%	n	%	n	%	n	%	n	%	n	%	n	%	n	%
Material	3	8.3	5	13.9	0	0.0	0	0.0	1	3.7	0	0.0	0	0.0	0	0.0	0	0.0	1	4.8	0	0.0
Fit	2	5.6	2	5.6	1	3.4	0	0.0	1	3.7	0	0.0	0	0.0	0	0.0	0	0.0	1	4.8	0	0.0
Ease of use	2	5.6	2	5.6	0	0.0	1	4.3	1	3.7	0	0.0	0	0.0	0	0.0	0	0.0	1	4.8	0	0.0
Size	1	2.8	2	5.6	0	0.0	1	4.3	1	3.7	0	0.0	0	0.0	0	0.0	0	0.0	1	4.8	0	0.0
Wash durability	1	2.8	2	5.6	0	0.0	0	0.0	1	3.7	0	0.0	0	0.0	1	11.1	0	0.0	1	4.8	0	0.0
Style	1	2.8	2	5.6	0	0.0	0	0.0	0	0.0	0	0.0	0	0.0	0	0.0	1	9.1	1	4.8	0	0.0
Quality	1	2.8	2	5.6	0	0.0	0	0.0	1	3.7	0	0.0	0	0.0	0	0.0	0	0.0	1	4.8	0	0.0
Other	1	2.8	1	2.8	1	3.4	0	0.0	0	0.0	1	5.6	0	0.0	0	0.0	0	0.0	0	0.0	0	0.0

**The issues are ordered (top to bottom) from most frequently to least frequently identified across the duration of BT.*

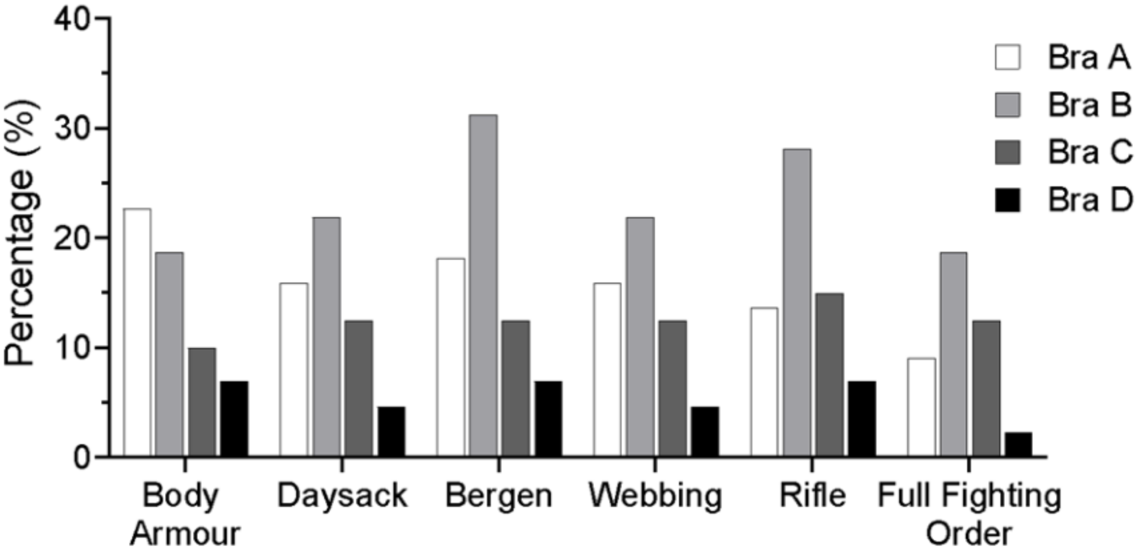

Online resource 24. Percentage of participants who found the four bras less comfortable when worn in combination with additional equipment (n = 54).

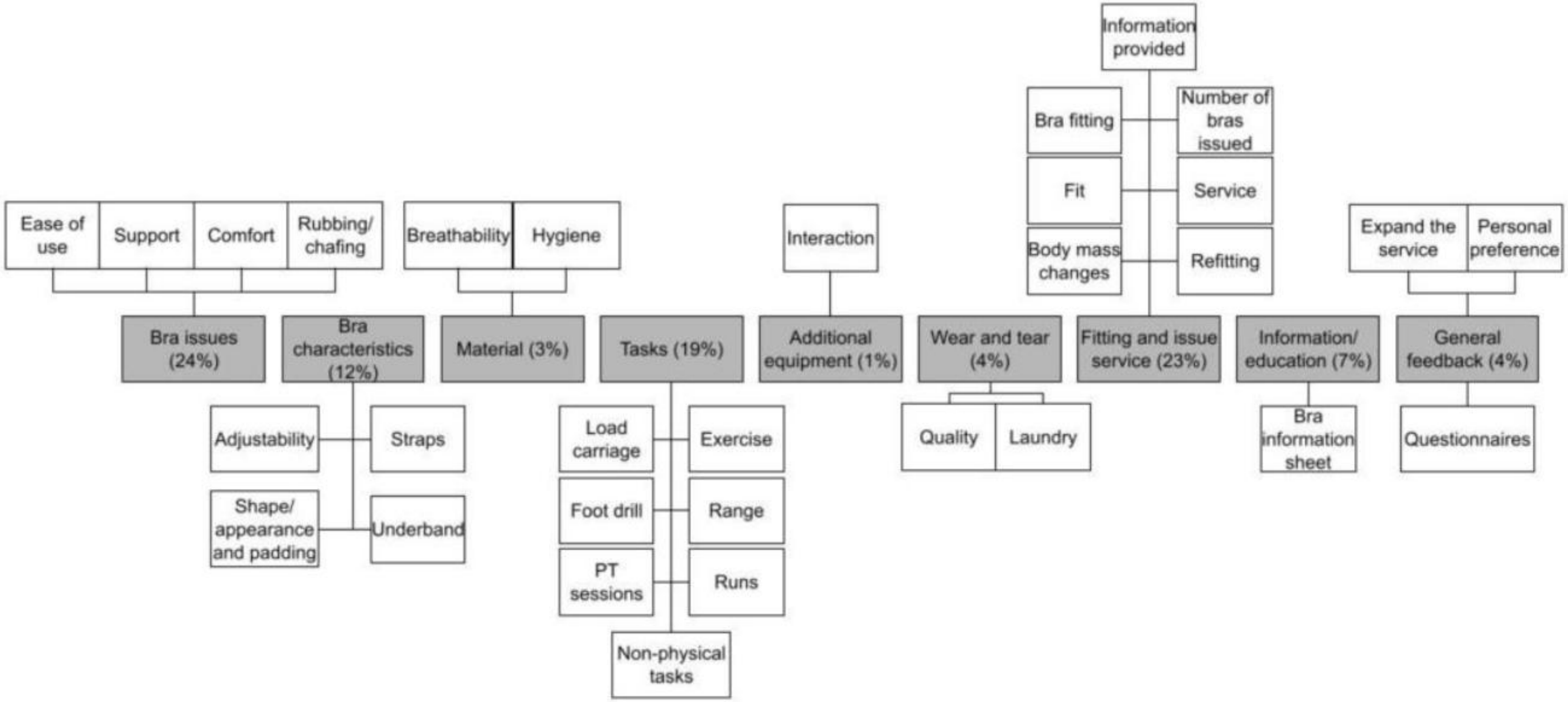

Online resource 25. Schematic of the 9 higher-order themes (grey boxes) and general dimensions (white boxes) identified from focus groups with Basic Training recruits at the end of training. Percentage (%) of comments within each higher-order theme out of the overall number of focus group comments made by recruits are presented.

Online resource 26. Number and examples of comments for each bra related to **the 'best' sports bra** higher-order theme.

Bra	fc	Example comments
A	10	"It's… comfortable, decent quality and padded. Just (a) nice bra to wear for everything."<br><br>"(I) prefer bra A because … I felt it had the most support, especially for when we were doing … battle PT and the more strenuous activities. I found it the most comfortable and most supportive to wear, but I could also use it in my daily routine if other (bras) were in the wash and I didn't have any issues with it and I was perfectly comfortable...."
B	2	"I think probably B for me because you can adjust the straps doesn't rub and (its) very comfortable …(you) don't know you are wearing it …easy to get on and off after you've done activity or before and after showers."
C	9	"Bra C (for) support and it was easy to put on like a normal bra. I didn't have to faff with putting it over my head after the shower and getting stuck to me."
D	12	"Definitely bra D for everything … it's so much more comfortable ... It doesn't really like move around or budge or anything like that."<br><br>"Bra D preferably, it's just … one single bit of material. It just seems a lot more comfortable for myself."<br><br>"I would marry bra D."

Note: fc = frequency of comments

Online resource 27. Number of comments and examples from each general dimension related to the **bra issues** higher-order theme.

General dimension	Issue	fc	Example comments
Ease of use	Top clasps on bras A & B difficult to do up alone	14	“Fiddly to try and get the latch at the top of bra A and B. I can do it (be)cause I'm flexible, but I've had to help couple of the lasses a few times. That’s alright whilst you’re here (in phase one), but when you go to phase two and you’ve got a room on your own, you can’t just go knocking about asking people ‘can you do my bra up’.” “You just can't reach … the clip”
	Top clasps on A & B take too long to do up	3	“…I'd have to get someone else to help me and that is such a pain when you have 15 minutes to get ready in the morning.” “When in this environment you don't know when they're (training staff) suddenly going to say you've got one minute to get out and then you don't know if you're going to get the strap on in time. So, I think that if I'm in a hurry I'm going to choose the easiest bra to (put on), but not necessarily the most comfortable for the next activity.”
	Donning techniques	4	“You didn’t have to undo the top clip on the bras, you can just whack it over your head and do the bottom clasp up” response from different recruit “I can’t do the bottom one, I can’t do it up behind me”
	Ease of use vs support	2	“(The) top clasp putting it on and like getting it on after the shower (was hard), but I wasn't really bothered because there was more support.”
	Bra D positive as no clips or clasps	2	“Bra D because it doesn't have … buckles.” “I like the speed and ease of use.”
	Bra C straps tricky to get on	2	“I had bra C and I did the cross back but it's a bit of a nightmare to get the back crossed and then put that over your head.” “I get someone else to do that (Cross the straps after donning).”
	Bra D difficult when wet	3	“Bra D can be difficult to get on or off when you're sweaty or you've been in the shower and you have to have a buddy system, to get it on and off sometimes so that can be interesting.”

			"The only thing bra D is bad for is after swimming. We've been getting change after swimming and someone would go I've made a terrible mistake and they're all tangled up in it."
	Bra C easy to don	1	"It was easy to put on like a normal bra I didn't have to faff with putting it over my head after the shower and getting stuck to me."
Comfort	Bra A Comfortable	7	"Prefer bra A because I found that the most comfortable………. I was perfectly comfortable wearing it whenever."<br><br>"Personal for me, it was bra A. It gave me the most support and was comfortable as well and really good for PT."
	Bra B comfortable	2	"Bra B was very comfortable you … don't know you are wearing it"
	Bra B uncomfortable	2	"The bottom elastic … really tight (it) tends to become uncomfortable."<br><br>"I wore bra B for field exercise and (I) didn't like it. It was not very comfortable."

Note: fc = frequency of comments

Online resource 27 continued.

General dimension	Issue	fc	Example comments
Comfort continued…	Bra C comfortable	5	"Bra C is a lot more comfortable."<br><br>"Bra C was really comfortable."
	Bra D comfortable	12	"It's … most comfortable and you … forget it's there sometimes … and I don't have to worry about clasps if I'm on my own, especially on field exercise I'd wear that one (D) because it's the comfiest."<br><br>"Bra D provided less support, but for comfort bra D was my favourite..."
	Prioritise comfort over support	6	"(Prioritise) Comfort because it's easy to move around (in) and when you go to sleep … it feels like you haven't got one on."

	Comfort and support come hand in hand	2	"I've always grown up wearing sports bras. I don't really wear a normal bra, so I find comfort in sports bras, so I find it supportive wearing a sports bra and I find it comfy as well."
Support	Bra A Supportive	8	"(I) prefer bra A because the I felt it had the most support especially for when we were doing … battle PT and the more strenuous activities."<br><br>"Bra A gave me the most support, was comfortable as well and really good for PT. Give me so much support."
	Bra B Supportive	3	"I think bra B, I found the most supportive for … running and things."
	Bra B Unsupportive	4	"Bra B … I don't feel supported with the fabric. "<br><br>"Bra B does not support me in any way, shape or form. I did a push up and my boobs fell out in a PT session."
	Bra C Supportive	5	"I thought it was very supportive."<br><br>"Bra C was most supportive, especially when I changed the straps (to cross over) for extra support."
	Bra C Unsupportive	1	"I just don't feel very supported in it."
	Bra D Supportive	1	"But that's because I've got really small boobs, so I don't need a lot of support"
	Bra D Unsupportive	4	"It's got no support, it's the comfiest one, but it's got no support. It's not tight enough…"
	Better support than personal bra	1	"I wore mine (own bras) less actually because these ones were more supportive."
Rubbing/Chafing	Bra A	7	"Bra A do(es) rub when I'm doing drill."<br><br>"I've got quite a lot of chafing from that bit (edge of cup), especially when (your) arm would rub up against it … if I wore it for drill. The first time I wore it was a drill session and the rubbing from that quantity of movement was bad."
	Bra B	1	"I got a lot of rubbing."
	Bra C	7	"The plastic (front adjustor). it just digs in every time I wear bra C. It digs in and I have a red mark that it rubs on."

			“Bra C rubs when I'm doing drill.”

Note: fc = frequency of comments

Online resource 27 continued.

General dimension	Issue	fc	Example comments
Rubbing/Chafing continued…	Bra D	3	“I found bra D quite difficult to wear for things like marching and drill because I'd end up with underarm chafe.”<br><br>“I don't wear it because even marching just to the scoff house, it would start rubbing bad.”
	Unavoidable Rubbing and chafing	7	“It's not preventable chafing though. Obviously, some bras chaff less than others. … but no matter what we do here, we're marching everywhere ... So, you're going to chafe, the boys’ chafe and they don’t wear bras, it's inevitable.”
	Location – Lateral panels/Edge of cups	7	“The extra seam where they're so chunky compared to the rest of the fabric, they stick out. So as your arm goes past it just catches it.”<br><br>“…Because you've got an extra bit of fabric there, (and the action) your arms are pinned quite close (to the side). … and it's all the friction of the material and the skin isn't the best, and we march everywhere, so it's constant.”
	Location - Underband	2	“On the underband.”

Note: fc = frequency of comments

Online resource 28. Number of comments (fc) and examples from each general dimension related to the **bra characteristics** higher-order theme.

General dimensions	Issue		fc	Example comments
Adjustability	Wanted adjustment		5	“I do like how adjustable it is.…once I've adjusted mine, I can just throw them on quickly, but (its) just getting it right the first time can take 10 minutes.”<br>“I think adjustment definitely (for) A and B was a must, but once you've adjusted it, you don't really have to do it again. I think when I first put it (bra C) on I adjusted and I haven't changed it since then.”
	Didn’t want adjustment		1	“It's just easier when they rush you around instead of faffing around with stuff (e.g. clips) and this (bra D) it's easier.”
	Easy to adjust		2	“They (are) all very easy to adjust and once you know that you can adjust (its) pretty straight forward.”
	Difficult to adjust		3	“None of the sports bras are easy to adjust the straps on, not even bra C.”
	Lack of knowledge about how to adjust		5	“Bra A I didn't realise until … last week that you could adjust these bits (straps) so it was always quite tight to get on and off but now it's perfect.”<br>“When we were at the fitting the lady that did it with me was very thorough, she did it really well…, but she did adjust it herself. So obviously …I knew she was adjusting (but) I didn't realise how much by or how to or where to adjust it. So then when they arrived … on the default …you just think, oh, well, it's been fitted. So …I didn't even think (I needed to) … Bra C, that's just like a normal bra so everyone knows how to adjust a normal bra where as these (A and B) I had no idea.”
Straps	Coming undone		5	“They just ping(ed) off when I was wearing it.”<br>“The straps keep coming off the top clasp and those ones on the side as well.”
	Digging in		2	“The plastic thing (on front of the strap), it just digs in every time I wear bra C … and I have a red mark.”
	Thick straps		4	“Maybe wider straps with more padding, bra A was my favourite when I had my webbing on (be)cause it's just that little bit thicker.”<br>“I like a thick band going over my shoulders.”
	Bra A	+ve	1	“Bra A for me personally that covered everything, size, shape and the way it looked.”

Shape/ Appearance and padding		-ve	1	"Because it's a fixed shape, there's nothing you can do about the fact that it gapes."
	Bra B	+ve	0	
		-ve	2	"I put bra B on and it wasn't even the way it felt I just felt like Bridget Jones. It feels like a … granny bra in the way it looks and …it made my boobs look pointy."<br><br>"The material was just too thin, you feel a bit too exposed. I still want my underwear to look nice, even though it's just me wearing it."
	Bra C	+ve	2	"I like bra C, the way it looks on me, more perky in a way."
		-ve	5	"Bra C the cups were unnatural for me, it just did not look like your average breast shape…. I found it too pointy for me."<br><br>"It gives me pointy shaped boobs. It sounds really weird, but. I don't have pointy shaped boobs and it just gives me them for some reason."

Note: fc = frequency of comments

Online resource 28 continued.

General dimensions	Issue		fc	Example comments
Shape/ Appearance and padding Continued …	Bra D	+ve		
		-ve	2	"Bra D because it's got no shaping to it, I don't like bras like that. Personally, I'd always wear my own bra underneath … to, keep some shape and it would hold it more firmly for me."
	Padding for modesty		11	"Padding is quite important just to cover like the … nipple shape because you just don't want them hanging out."<br><br>"If you're not wearing a padded bra and you've got just the green T-shirt on your nipples are very obvious. Especially if we're out(side) and It's cold or there's a bit of a breeze … you feel really … conscious because it's very prominent."

	Padding for support	1	"I like padded bras because I feel a lot more supported."
	Padding for protection	2	"Bra A, … it has the extra padding. So, if you're, jumping down to prone or if you're, slinging a rifle across (you) there's a bit of added protection."
	Did not want padding	5	"I don't like things with padding on … I've experimented with it in civi street and I've (decided) that's not for me."<br><br>"I just didn't like (the) padding."
Underband	Too tight (bra B)	2	"Only bra B even after loosening it …the underband was excruciating… it was just so uncomfortable I couldn't think of anything else."<br><br>"(The) bottom elastic it's really tight it tends to become uncomfortable."
	Too tight (Bra D)	1	"It's that style of bra that I've tried in general. I just feel I can't breathe. Its fine on my boobs because I have relatively small boobs …. I just struggle with feeling like I've got enough room around the underband and my rib cage."

Note: fc = frequency of comments

Online resource 29. Number of comments (fc) and examples from each general dimension related to the **tasks** higher-order theme.

General dimension	Issue	fc	Example comments
Field exercise	Comfort is priority	9	"I wore bra D for battle camp, … because I find it comfy. "<br>"Comfort …because it's easy to move around and when you go to sleep …it feels like you haven't got one on."
	Bra C good compromise of comfort and support	3	"I preferred wearing bra C and crossing the back for battle camp."<br>"I want both (support and comfort) on battle camp, that's why I went for C."
	Sleeping in Bra	4	"Bra C you can sleep in and it doesn't have any wires."
	Do not take bra off	4	"You don't really take your bra off."<br>"I know it's a bit gross, but I actually didn't take it off."
Tabbing	Shoulder straps dig in	1	"The way the webbing sits right on your shoulder and then Bergen on top is a lot of pressure, and then they dig in."
	Reliability	1	"Wore bra D, if I knew I was doing a major tab or anything, just because its reliable."
	Bra A – More support	2	"Bra A … I've got a slightly larger chest so I don't get support off any anything else."<br>"I would wear bra A, even though it's a bit more awkward to put on, it's more supportive so I'd put that one on for something more intense."
	Bra A&B back clip	2	"I wore bra A and … it was pulling at my shoulders too much because of the clip at the back."
PT sessions	Bra A	9	"If we're going off PT, I'll probably wear bra A because it's a lot more supportive."<br>"(I) prefer bra A because the I felt it had the most support. Especially for when we were doing … battle PT and the more strenuous activities."
	Bra C	8	"Bra C for high impact PT."

			"Bra C is good for circuits and stuff."
	Bra D only for weights	4	"The only workout you'd be able to do with bra D is probably weights because you're not running around."
Range activities	Padding	3	"I wore bra A and C, just cause it's padded."<br><br>"Lying down on your chest you just want cushioning."
	Low support	1	"I don't need that much support because it is just very slow movements."
Runs	Cannot run in Bra D	3	"Would not wear sports bra D for running."<br><br>"There is no way I could wear bra D (for running)."
	Bra B is most supportive	1	"Bra B I found the most supportive for running."
Foot drill	Need less support	6	"Probably go (for) the less supportive ones, (I) don't think it needs to be supportive."<br><br>"Just wear a little sports bra from Primark."
	Did not wear sports bras	4	"I wear a bralette just from Primark… £2.50."<br><br>"I don't wear sports bras for foot drill."

Note: fc = frequency of comments

Online resource 29 continued.

General dimension	Issue	fc	Example comments
Foot drill Continued…	Chafing	6	"Probably the worst thing for chafing (be)cause It's constant rubbing. Especially on the side (of the) armpit. You need a soft material."<br><br>"I've got quite a lot of chafing…especially when your arm would rub up against it … when I wore it for drill"

	Rifle drill	4	“When I've been doing weapons drill, I've been wearing bra A for comfort because (you are) slamming into the shoulder all of the time.”<br><br>“Rifle drill poses a whole different question with the bras because you're having to rest it on your shoulder. For me …the rifle was pressed on the strap and strap was digging in … it's marked, bruised and rubbed it.”
Non-Physical tasks	Bra A	1	“A or C probably because there's no doubt I’d end up having to run somewhere anyway, so I was just being prepared.”
	Bra B	6	“B for classroom. I wear it in classroom for breathability and because when it's hot … you can feel the still feel the breeze.”<br><br>“After I've had a shower … I just chuck on bra B to go to scoff or just to wear around ironing.”
	Bra C	4	“So, if we'd seen the timetable and it was a more classroom based my go to for that was bra C because I didn't need the heavy duty straps.… I could use it as my go to. Yes, I might be marching around, but most of the days in the classroom, so it's quite chilled.”<br><br>“Mine was bra C for that kind of day because I found that one really comfortable and it was supportive enough if they sent us around the block. It would still be better than an everyday bra.”
	Bra D	13	“If I knew that we were going to have, a day of briefs and lessons, I'd wear bra D just (be)cause it's comfortable (and) It's quick and easy to put on.”<br><br>“Bra D that was more comfortable so I put that on for a day ….we weren't doing anything.”
	Normal Bra	4	“I’d just wear a normal bra.”<br><br>“I have a gym shark low cut bra I wore, and then I've switched between that and just normal bra.”

Note: fc = frequency of comments

Online resource 30. Number of comments (fc) and examples from each general dimension related to the **additional equipment** higher-order theme.

General dimension	Issue	fc	Example comments
Equipment interaction	Rubbing is inevitable	2	"Any bra I wear digs in. I sometimes wore my own sports bras that I bought and even then it would just dig in and I'd have to move stuff around… It's not the bras fault. It's definitely the webbing and then the Bergen."<br><br>"I feel with wearing Bergen's and webbing. You could wear any bra with it and it would rub."
	Thicker straps	2	"I prefer bra A because the straps are thicker and it takes a little bit of the webbing off my shoulders. I feel it's just a little bit more cushiony on the shoulders."
	Top clasp digging in	1	"The only problem with A is if you put something on your back, that clasp digs in. Body armour was all right but a day sac or a Bergen, (left a) little red square on your back."
	Comfort with Body Armour	1	"I would say I went for comfort because we wear body armour that almost like having a corset. So that was my support, so I needed comfort for underneath."

Note: fc = frequency of comments

Online resource 31. Number of comments (fc) and examples from each general dimension related to the **material** higher-order theme.

General dimension	Issue	fc	Example comments
Breathability	Sweaty Boobs	3	"Wicking material on the bands …to stop you sweating."<br>"They've squashed my boobs together and I get really sweaty, there's a lot of grossness so it would actually be nice to have a little bit of separation."
	Bra A Good	2	"I feel like bra A was the best for me at not getting sweaty…I felt it was more breathable."
	Bra B Good	2	"I find it very airing if I've had like a sweaty day and I'm just going to scoff or something I'll put that on because it's very lightweight and it just feels airy."
	Bra C Good	3	"Bra C was really good at not putting the sweat anywhere else."
	Bra D Bad	2	"My Nike sports bras are similar to this design. (But) feel like sweat wicking material, whereas this one probably is OK if you just wear it in the block or not doing anything too strenuous."<br>"One thing I will say about this one (D) is … It does give me sweaty boobs, it's not the most breathable."
Hygiene	Rash	2	"I've had a rash between my boobs, and I get a rash, little spots in the middle."
	Exercise	3	"You collect sand and stuff and you shake it out. But then your boobs get squashed together and it looks weird when you're there shifting a boob left and right to get it out."<br>"Because it's been sweaty it's been grim you end up with bugs when you go for your shower on the last day and you found bugs and leaves and all sorts that have just accumulated in there."

Note: fc = frequency of comments

Online resource 32. Number of comments (fc) and examples from each general dimension related to the **wear and tear** higher-order theme.

General dimension	Issue	fc	Example comments
Quality	Good quality bras	3	“Quite good quality stuff.”<br><br>“There good quality.”
	Elastic going	3	“I think obviously elastic goes and things, but it's going to happen.”
	Disintegrated	1	“They lasted to the 14th week. It was good and I used it regularly… but the whole mesh has thinned out and split so I can no longer wear it.”
Laundry	Shrunk in the laundry	4	“When you're here they put things in the dryer, so they shrink quite a bit and it gets quite tight… but you don’t really get to say if you hang it up or put it in the dryer.”
	Not having access if bra in the wash	6	“I personally don't mind putting on a really stinky bra if I need it for a tab, because I think having a good bra gives me a good mindset.”<br><br>“That's the only problem with it…if say on Saturday we had battle PT, we've come out soaked. … the next week, we weren't having a wash till Thursday so then Monday, Tuesday, Wednesday if we've got a tab we can't wear that.”
	Don’t wash bras regularly	5	“They’re getting washed about once a week with us That’s so bad when you think about it.”<br><br>“I probably don't wash it as much as I should.”

Note: fc = frequency of comments

Online resource 33. Number of comments (fc) and examples from each general dimension related to the **fitting and issue service** higher-order theme.

General dimension	Issue	fc	Example comments
Bra fitting	Never been fitted before	7	"I think it's good like cos I've never been measured before."<br><br>"I've never had a bra fitting … just growing up with dad would be a bit awkward about it. So, no never did (have one). … It was good, it wasn't awkward at all, it was very professional, very quick as well and quite accurate for me."
	Didn't know bra size	2	"I didn't know my bra size. Whenever I go shopping I just see whatever bra there is, and I'll just go yeah, that'll fit, I'll take it."
	Positive experience	12	"…It's so much comfier and I think it's just how you tighten the straps and everything. I think it's generally helped me"<br><br>"I found it really useful especially with the way you have sister sizes for bras…some of the bras I was a different size and I think it was still the sister's size, but it fitted better in that style of bra so that was useful."
	Negative experience	2	"When I went in …they (asked) what size (I was)? I said I've lost a lot of weight since I've had a bra fitting, so … I just wear my sports bras. I've actually haven't been fitted for a normal bra in ages. (They asked) what size were you? … I think it was 34 DD? (She said) Yeah, that's about right. …I think they give me a 34D in the end but there's no way my back is still a 34, I think I'd lost about 5 stone. …OK, whatever so I just took it."<br><br>"I think I was expecting to be actually measured with the tape like you said because prior experience and stuff, not just asked what size are you? And then yeah, there's a bra let's just have a look."
Fit	Bra B cups too loose	3	"Wearing bra B because the extra material around the cups it didn't support me properly."<br><br>"It was just a bit saggy in the cup… It wasn't filling it and it just seemed a bit pointless."
	Underband too tight	3	"Bra B I've adjusted … I think it's just my body shape, but around the underband it's just too tight."<br><br>"Bottom elastic it's really tight. Tends to become uncomfortable."

	Bra C straps too tight	1	"When I wore bra C and I crossed it over, I felt quite uncomfortable it was very tight on me, even if it was like the longest setting."
Refitting	To resize due to fit	11	"If I got measured again, I'd be a different size 100%."<br><br>"Then it's absolutely pointless its been in my locker since week three cos it doesn't fit anymore"
	To exchange/select new bras	7	"If we'd had, a re-fit halfway through so we could either change the ones we've got or, pick new ones to take."<br><br>"Two of them, I had to cancel out. …But when they do a refitting maybe exchange … if bra B because I can't wear it…it's just sat in my draw it's just a waste of a bra."
	Timing suggestion:<br><br>Week 4<br><br>Week 6<br><br>Week 7<br><br>Week 8	 <br>1<br><br>3<br><br>8<br><br>2	"I'd say mid-way 7 week"<br><br>"Maybe halfway through, weeks 7-8?"<br><br>"Halfway through because it's the first, 5-6 weeks where you're really active and you lose the most weight after exercise..."

Note: fc = frequency of comments

Online resource 33 continued..

General dimension	Issue	fc	Example comments
Body mass changes	Change in body shape/size	11	"… I might not have lost weight while I've been here, but my body has changed completely. I've completely lost my boobs since we've been here."<br><br>"I lost four or five kg, so my tits just went (Action to represent shrink) so then certain bras didn't fit me."
Number of bras issued	4 was enough (If all were worn)	7	"I was going to say because there's four you can just keep switching and all of them work well on different activities."<br><br>"I think if you wear them all, it's probably enough"

	4 was not enough (if you didn't wear them all)	8	"I think, if you only had ones that were issued. I think you'd struggle…You're never going to find all four are appropriate. You're always going to pick one, maybe two, the best case around three of them."<br><br>"I think that four was enough but if you only wear two, then you're a little bit short."
	Additional own bras used	7	"I think I brought seven of my own sports bras because I didn't know I was going to get these until they said you're going to a bra fitting … so obviously at the beginning we were doing phys, at least five days a week or similar…sometimes twice a day …. so it was good for me, but I'd mix them with my own."<br><br>"I switched between my own bra and these bras, so I'm pretty happy with the amount that I have."
	Washing availability	4	"The last week we had three tabs in one week I would always wear bra A for a tab, so when it needed washing, I couldn't wear it."
	Ideal number	3	"I would say 4 is the minimum rather than anything else."<br><br>"Especially with the physical activity that we do, not saying we need 30, but we need at least one for every day...."
Information provided	Kit list	4	"I bought 5 from home because that's what they said on the kit list."<br><br>"We all got a different kit list and mine did not specify how many to bring."
Service	Did not fit when bras arrived	8	"They fitted perfect(ly) at the fitting and when I got them, they just didn't fit. I don't think my boobs shrank that much in that little amount of time"<br><br>"I didn't actually wear any of the bras because they didn't fit. ……. You said to speak to our section commanders, our female section commander wasn't in for ages and obviously the males are uncomfortable and didn't know what to do about…… I think I did go to speak to him but then I changed my mind because …they're not going to be able to do anything about it."
	Walk away with bras on the day of fitting	2	"That would be more beneficial for us"<br><br>"It might be better if went to the fitting and we got to walk away with the ones that (we) tried on there."
	Speedy delivery	6	"They arrived really quick. It was before we had any PT."<br><br>"This speed was quite good I'd say, it came pretty quickly."

	Extra bras	2	“I actually got given an extra three bras very randomly about week four or five. I've left them in the package. We got given an extra 3 out of the blue and no one else did.”
	Did not wear	1	“I didn't really wear any of them just (be)cause I tried them and I thought my own was comfier.”

Note: fc = frequency of comments

Online resource 34. Number of comments (fc) and examples from each general dimension related to the **information** higher-order theme.

General dimension	Issue	fc	Example comments
Bra Information sheet	Did not read	14	"You get that much paper when you here, on a daily basis that you lose everything."<br><br>"No but honestly, coming into it, this whole experience was so overwhelming that I can't even reply to my family's messages … so I think for me, it was not on the priority list. I didn't realise how necessary it was. I would look at the bra and go that looks like it's high intensity I'm going to wear that today. I'm sure it probably would have been quite helpful, but I think I kind of did it off instinct."
	Looked at once	8	"I have read it, but I didn't follow it … I think you just go off what you find is more comfortable."<br><br>"I read it when you gave it to me, It's probably at the bottom of my locker somewhere."
	Followed it at the beginning	6	"I …learned what was right for me rather than what the sheet said."<br><br>"We put it on the notice board in our room for the girls who were using it and I'd look at it if I knew that we were having, a certain sort of day. I think because a lot of the stuff we've done we've never done before, so we didn't really know what would be the best support to wear during that activity it's nice that these bras had … notes at the side, it's good for this … Then we moved rooms and lost it."
	Suggestions:<br>Video<br>Sent out with questionnaires*	 4<br>3	"I think with today's generation of phones and I'm going to sound like such a boomer, but yeah more visual I'd say."<br><br>"In person because then we have to sit down and we have to listen to it. If you give us a piece of paper or a video during our own time. I'm not going to lie it probably won't happen."
	Not told what activities they were doing	2	"When we had PT sessions, we didn't know what the PT sessions were going to contain so it was just a guess of, I'll put this bra on and see if we're doing any of this and just try to wear the bra with the most variety at first… We would only know if it's tab, run, swim or general PT."

Note: fc = frequency of comments

Online resource 35. Number of comments (fc) and examples from each general dimension related to the **general feedback** higher-order theme.

General dimension	Issue	fc	Example comment
Expand the service	Phase 2	2	"I think that would be helpful in phase two, because the phys gets it's different and it gets harder."<br><br>"I think those will be doing heavier loaded marches in phase two. So, it would be good to get refitted."
	Wider field army	2	"Or even the women that have already been serving for years, a lot of our training team were saying, Oh my God, you get three sports bras! That's great I need new sports bras … not only recruits need sports bras."<br><br>"There might be women just on normal camps that … could really use a good sports bra, but have no idea what to pick."
Personal preference	Everyone is different	3	"Personal preference and everyone's going to be different."<br><br>"But the fact you two actually wear bra B baffles me…… The fact that you find that (D) the comfiest baffles me I can't breathe……. It just shows that we've all got boobs but we are so different."
	Safety	3	"I've always stuck to Nike or Adidas sports bras very similar to bra D, so I'm just sticking to what I know."<br><br>"You're coming into a new environment with lots of new things actually your bra was the last thing you wanted to think about."
Questionnaires	Timing	4	"It was good as well that you text us on the Friday because then the weekend we actually got free. So, we actually got time to do it, but if it was texting on Monday I'd just forget. So, it was good that we got that on Friday because then we actually pay attention."<br><br>"With timings of stuff obviously it's not your fault but …when it comes out and we're on exercise and then we come back we have to sort our kit we have to do this and do that and then when it comes to then having that free time, the next questionnaire is already out."

	Length	5	"If you sat there and just did it about 2 minutes, whereas If you made it longer, I think people would just get bored of doing it."
	Reminder	2	"The reminder, was helpful cos I always forgot."
	It was quite negative	1	"There was nothing to… put any positives on there. It was why didn't you wear it? Not why did you wear it."

Note: fc = frequency of comments
